# Therapeutic efficacy of nanoparticles and routes of administration

**DOI:** 10.1186/s40824-019-0166-x

**Published:** 2019-11-21

**Authors:** Dhrisya Chenthamara, Sadhasivam Subramaniam, Sankar Ganesh Ramakrishnan, Swaminathan Krishnaswamy, Musthafa Mohamed Essa, Feng-Huei Lin, M. Walid Qoronfleh

**Affiliations:** 1 Department of Microbial Biotechnology, Bioprocess and Biomaterials Laboratory, Bharathiar University, Coimbatore, India; 2 Department of Extension and Career Guidance, Bharathiar University, Coimbatore, India; 3 Department of Food Science and Nutrition, College of Agricultural and Marine Sciences, Sultan Qaboos University, Muscat, Oman; 4 Institute of Biomedical Engineering and Nanomedicine, NationalHealth Research Institutes, Miaoli, Taiwan; 5 Research and Policy Department, World Innovation Summit for Health (WISH), Qatar Foundation, P.O. Box 5825, Doha, Qatar

**Keywords:** Nanoparticles, Nanocarriers, Drug delivery, Drug administration, Targeted drug delivery, Administration route, Therapeutics, Cancer

## Abstract

In modern-day medicine, nanotechnology and nanoparticles are some of the indispensable tools in disease monitoring and therapy. The term “nanomaterials” describes materials with nanoscale dimensions (< 100 nm) and are broadly classified into natural and synthetic nanomaterials. However, “engineered” nanomaterials have received significant attention due to their versatility. Although enormous strides have been made in research and development in the field of nanotechnology, it is often confusing for beginners to make an informed choice regarding the nanocarrier system and its potential applications. Hence, in this review, we have endeavored to briefly explain the most commonly used nanomaterials, their core properties and how surface functionalization would facilitate competent delivery of drugs or therapeutic molecules. Similarly, the suitability of carbon-based nanomaterials like CNT and QD has been discussed for targeted drug delivery and siRNA therapy. One of the biggest challenges in the formulation of drug delivery systems is fulfilling targeted/specific drug delivery, controlling drug release and preventing opsonization. Thus, a different mechanism of drug targeting, the role of suitable drug-laden nanocarrier fabrication and methods to augment drug solubility and bioavailability are discussed. Additionally, different routes of nanocarrier administration are discussed to provide greater understanding of the biological and other barriers and their impact on drug transport. The overall aim of this article is to facilitate straightforward perception of nanocarrier design, routes of various nanoparticle administration and the challenges associated with each drug delivery method.

## Background

### Nanotechnology and nanoparticles

**Fig. 1:**
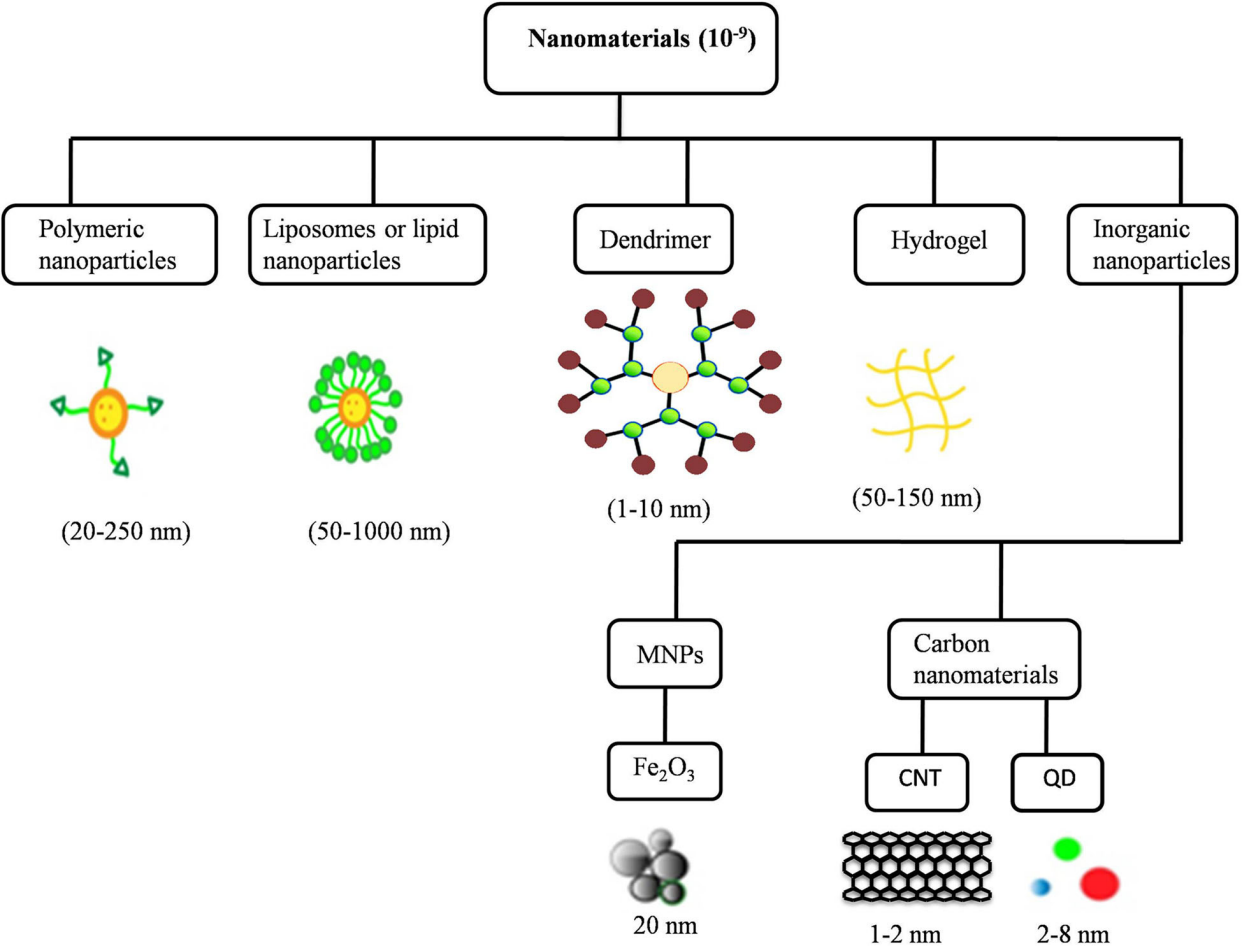
Various types of nanomaterials and their morphological features

In the Greek language, the words nano means “dwarf” and the SI prefix denotes 10^− 9^ or 0.000000001. By definition, nanotechnology is a fusion of advanced manufacturing science and engineering where the synthesis or assembly of material is aimed at the nanometer scale (1–100 nm) or one-billionth of a meter. The unique property of nanosized material as compared to bulk material is the advantage of more surface to volume ratio. Nanoparticles (NPs) have wide-spread applications in various sectors ranging from agriculture to medicine. In medicine, nanoparticles are continuously being improved for drug delivery, screening of various diseases and tissue engineering, to name a few. Consequently, nanotechnology has begun playing a pivotal role in catalysis, energy and environment, agriculture, optics, sensors, computers and many others [1]. The current review explores the advancements in nanoparticle-mediated targeted drug delivery along with discussing the efficacy and limitations of various administration routes. Besides conventional drugs, recombinant proteins, vaccines, and nucleotides may also be effectively delivered by NPs [2]. Nanoparticles can be synthesized from various organic or inorganic materials such as lipids, proteins, synthetic/natural polymers, and metals [3, 4]. Nanoparticles can be classified into several groups such as polymeric nanoparticles, liposomes, dendrimers, micelles and inorganic nanoparticles, based on the components used for synthesis or the structural aspects of the NP (Fig. 1). The fabrication methods and the properties of nanoparticles would also determine its application and utility. However, the type of nanoparticle used in the targeted delivery of therapeutics has its own positive and negative influences [3].

### Natural and synthetic polymer nanoparticles

A wide range of polymer nanoparticles has been described owing to advancements in polymer science and nanotechnology. The unique property or desirable characteristics of polymeric nanoparticles decide its potential application. The most important properties of polymeric nanoparticles are biocompatibility and biodegradability. Therefore, they are widely used as a drug delivery system [5]. Besides, they must retain high stability in a biological environment. For drug delivery applications, the drug may either be encapsulated or immobilized on the polymer and subsequently released into the target site by diffusion or desorption [6]. Based on the drug-encapsulation method they are classified into three types. The first type consists of linear polymers (i.e., it uses a covalent approach for drug conjugation), the second category is labelled as polymeric micelles (formed by amphiphilic block copolymers) and the third group involves hydrogels (i.e., hydrophilic drug encapsulation) [7]. The main characteristic of the polymeric nanoparticle is the controlled release of therapeutic agents. Biodegradable polymeric nanoparticles are not only used as carriers for pharmaceutical drugs but also to deliver proteins and DNA. Synthetic polymers such as polylactide–polyglycolide copolymers, polyacrylates and polycaprolactones (PCL), polylactic acid (PLA), poly (lactic-co-glycolic acid) (PLGA) are often used in nanoparticle synthesis. The tissue compatibility nature of PLA and PLGA make them useful in controlled release formulation for parenteral and implantation drug delivery applications [8]. The structural properties of polysaccharide nanoparticles are determined by their chemical composition [9].

Polysaccharides are a substantial component of natural polymers and are mainly derived from algae (e.g., alginate), plant (e.g., pectin & guar gum), microbial (e.g., dextran & xanthan gum), and animal (chitosan& chondroitin) products. Synthetic polymer nanoparticles are preferred over natural polymeric nanoparticles for sustained release [10]. These polymers have exceptional material properties because of their chemical structure and type of functional group(s). Moreover, they can also be altered based on the method of synthesis. The advantages of synthetic polymeric nanoparticle include easy fabrication and absence of biological contamination. Polycationic polymers have shown better mucoadhesive properties and, as a result, are widely used in mucoadhesive drug delivery [11]. Chitosan is mucoadhesive and soluble only at acidic pH. Hence, chemical modification of chitosan is being carried out to enhance its mucoadhesive properties. Chitosan derivatives like trimethyl chitosan (TMC), thiolated chitosan, chitosan-ethylenediaminetetraacetic acid, etc. have showed improved solubility and mucoadhesive properties [12]. Sajomsang et al have synthesized two methylated derivatives of chitosan and found that increasing the degree of quaternization will lead to stronger mucin-particle interaction [13]. Thanou et al reported the ability of TMC to enhance the permeation of the peptide drug buserelin, a gonadotropin-releasing hormone agonist, across intestinal epithelia in vitro (Caco- 2 cell monolayers) and in vivo (rats) [14]. Gatti et al prepared nanoparticles based on chitosan/dextran sulfate formed by polyelectrolytes condensation for insulin delivery. The encapsulation prevented insulin from partial degradation and displayed sustainable release indicating efficient mucus complexation between mucin and nanoparticles [15].

Polymer-coated nanoparticles have been used to improve the biodistribution kinetics. The nanoparticle surface coated with polyethylene glycol (PEG) has increased blood drug concentration in the brain, kidney, and intestine by evading the reticuloendothelial clearance system [16]. The bio-inert characteristic of the PEG polymer is a classic example of the preparation of cytocompatible multifunctional polymeric nanoparticle and surface modification. The foremost desirability of PEGylation for drug delivery lies in its ability to extend their stability in the mucous and to reduce the nanoparticle clearance by the immune cells [17]. The unique architecture of nanosized carriers considerably overcomes the limitation of conventional drug delivery methods and has an impact on advanced therapy for various diseases like tuberculosis and pulmonary hypertension [3].

#### Poly (lactic-co-glycolic acid) (PLGA)

Among the synthetic polymers, poly (lactic-co-glycolic acid) PLGA (obtained by the condensation of lactic acid and glycolic acid) is considered a base material for numerous biomedical applications. The main appeal of PLGA NPs can be attributed to the fact that they are hydrolyzed into their monomeric units such as lactic acid and glycolic acid, which are byproducts of various metabolic pathways in the body under normal physiological conditions [18]. Technological sophistication has enabled PLGA nanoparticles to be explored not only to encapsulate anticancer drugs, diabetic medications or hormones but they also offer a platform for multifunctional imaging in cancer diagnostics [5]. One of the lures of using PLGA in medical devices (e.g., orthopedic fixation devices) or nanoparticle fabrication is that the rate of biodegradation can be controlled by adjusting its molecular weight (MW) or copolymer ratio [19]. The US Food and Drug Administration (FDA) and the European Medicine Agency (EMA) have permitted the use of PLGA for drug delivery applications in humans [20]. Despite PLGA having minimal toxicity, their acidic nature does not favor the release of acid-labile drugs. However, it could be revamped by formulating a suitable mix with carbohydrate polymers like chitosan, alginate, and poly (isoprene), etc. The combination of hydrophobic or amphiphilic polymers such as PLGA and PLGA-PEG offers great promise in drug delivery, but the applied experimental conditions like sonication could affect the stability of the drug molecule encapsulated within. Encapsulation of a range of anticancer drugs, namely doxorubicin, paclitaxel, dexamethasone, cisplatin 5-fluorouracil and 9-nitrocamptothecin, have been reported as using PLGA nanoparticles [21]. The PLGA microsphere has successfully protected the encapsulated DNA from nuclease degradation [22] and to attain a stable gene expression, the encapsulated DNA in PLGA has to undergo sustained release after intracellular uptake and endolysosomal escape [23]. To improve the efficiency of PLGA nanoparticle as a drug delivery system, zinc (II) phthalocyanine (ZnPc) was incorporated to increase the rate of permeation and tissue uptake for the photodynamic activity in mice [24]. Likewise, the functionalization of PLGA with polyethyleneimine (PEI) was shown to be effective in siRNA delivery. The existing evidence suggested that PLGA is one of the most successful in vivo biodegradable drug delivery systems owing to its simple hydrolysis degradation mechanism [24].

#### Chitosan

**Fig. 2:**
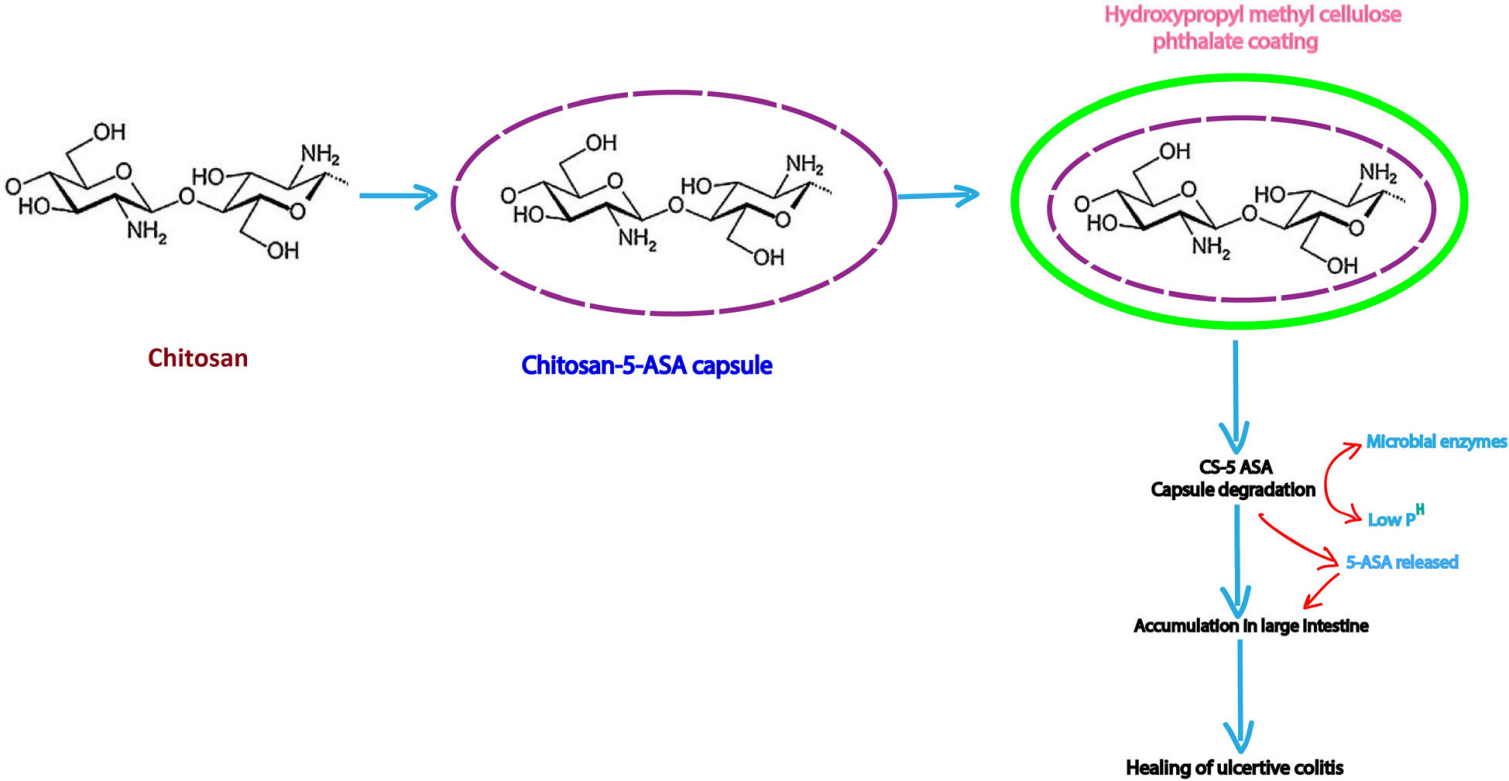
Chitosan based drug delivery. Chitosan containing 5-ASA capsules were coated with hydroxypropyl methylcellulose phthalate as an enteric coating material. After the oral administration of chitosan 5-ASA capsules, disintegration of capsules was assumed by microbial enzyme degradation along with the low acidic pH in the colon. Moreover, chitosan facilitate to stay 5-ASA in the large intestinal mucosa over a period of time and accelerates the healing of TNBS-induced colitis

There are numerous polymers that have been approved for biomedical applications. Among them, chitosan is the most important naturally occurring cationic polymer approved by the US FDA and EMA for tissue engineering, drug delivery and also gene delivery [25]. Mumper et al first reported the use of chitosan for in vitro gene delivery [26]. Hydrophobic polymers such as PLGA have a serious limitation in delivering macromolecules across the biological surfaces. Hence, colloidal hydrophilic polymers are the primary choice for delivering such macromolecules effectively. Through different mechanisms like ionic crosslinking or complexation and desolvation, chitosan is capable of forming colloidal nanoparticles which can protect the macromolecule of interest [27]. The excellent biocompatible and biodegradable nature of chitosan makes it useful in various drug delivery applications. The structure of chitosan is highly favorable for effortless functionalization with its primary hydroxyl and amino groups that also improve the physical and biological properties of chitosan during the conjugation process. The hydrophilic nature of chitosan aids an easy conjugation of hydrophobic moiety which in turn leads to the formation of self-assembled nanoparticles that are useful for targeted drug delivery applications [28]. Because of their effortless functionalization and mucoadhesive properties, chitosan-based delivery systems have been the most studied and demonstrated platform for delivering drugs or pharmaceuticals to various organs. Chitosan capsules were designed to enhance the localization of 5-Aminosalicylic acid (5-ASA) for colon-specific drug delivery (Fig. 2) [28, 29]. An affinity-based interaction between the hydroxyl and amino groups of chitosan and hydroxyl groups of dexamethasone has suggested that chitosan-films are useful for sustained release of dexamethasone [30]. Low molecular weight chitosan (LMWC) (19 and 31 kDa) are promising drug carriers (e.g., LMWC with prednisone) for renal or kidney targeting [31].

### Liposomes and solid lipid nanoparticles (SLNP)

The use of lipid-based nanoparticles was initially derived from the biocompatible concept, where the tiny lipid cholesterol molecules and phosphatidylcholine are popular [32]. Another reason for using lipid-based nanoparticles is their easy cellular uptake of drugs because of the outer lipid bilayer [3, 33]. Two of the most important lipid-based nanomaterials are liposomes and solid lipid nanoparticles. Liposomes consist of a lipid bilayer enclosing an aqueous core while lipid NP consists of lipid monolayer enclosing a solid lipid core [34]. While they are slightly different in their structure, both can be effectively used in drug delivery applications. Liposomes and solid-lipid nanoparticles are particularly considered effective in inhalation therapy for chronic lung diseases since they are stable during aerosolization [33]. The effectiveness of SLNPs was demonstrated by SLNPs loaded berberine (benzylisoquinoline alkaloid) which showed better bioavailability and increased the antidiabetic effect in a diabetic mouse model [35]. A phytocompound Aloe-emodin, an anthraquinone, loaded in SLNPs displayed increased anticancer effect in hepatoma and breast cancer cell lines [36]. Baeck et al noted increased bioavailability of curcumin in lymphatic cells when loaded with N-carboxymethyl chitosan-coated SLNPs [37].

### Dendrimers

Dendrimers are synthetic, well-defined and highly mono-dispersed symmetric molecules which have a repetitive branched pattern. They can demonstrate better physicochemical and rheological properties as compared to conventional linear polymers. Regardless of the advancements in dendrimer research, the use of dendrimers as drug carriers is still poorly translated into the clinical application [38]. Although it shows its excellence as drug and gene delivery agents, dendrimers can display cytotoxic and hemolytic properties, raising potential toxicity safety concerns. As dendrimers are non-degradable in the physiological environment, it results in serious side effects induced by the accumulation of non-degradable artificial macromolecules inside the cells or in the tissues. The cationic characteristics of these polymers result in an interaction with the negatively charged cell membranes, thereby causing cell destabilization with the leakage of cytoplasmic proteins and subsequent lysis [39, 40]. Moreover, the size and surface functionality of the final formulation is precisely controllable [41]. The surface of PEGylated dendrimers may have higher drug load than the unmodified dendrimers and is designed to escape the body’s defense actions and circulate in the blood for an extended period of time. A variety of drugs or therapeutic molecules can be encapsulated in dendrimers using a simple electrostatic interaction or covalent attachment [42–44]. The polyvalency and strong spatial distribution of multiple functionalities on the surface of the dendrimer are major assets making them a desirable agent for combating cancer, inflammation, HIV, etc. along with drugs and gene delivery [45]. The surface-modified dendrimers by lauroyl chains and PEG-2000 have significantly reduced cytotoxicity in Caco-2 cells [46]. Likewise, newer PEGylated polyamidoamine (PAMAM) dendrimers (4.0 G PAMAM) synthesized by Michael addition and amidation reactions were used for the delivery of the anticancer drug 5-fluorouracil which reduced the rate of drug release and hemolytic toxicity [47]. Acetylation of PAMAM dendrimers is reported to be a promising siRNA delivery agent again because of reduced cytotoxicity [48]. Several new dendrimers poly (propylene imine) (PPI), poly (amidoamine) (PAMAM), and poly(L-lysine) (PLL) (i.e., PEGylated PLL dendrimer with docetaxel) are in clinical trials owing to their well-defined architecture and facile surface tailoring [49]. These results substantiate a positive indication of dendrimers potential in nanotechnology-based cancer therapy.

Synthesis of multifunctional dendrimers for theranostic applications is a contemporary research direction. One of the emerging applications of dendrimers is focused on cancer theranostics. Differently sized macromolecular and nanosized dendrimer MRI contrast agents have been reported for various applications as they provide sufficient contrast enhancement [50–52]. The approved MRI contrasting agents are of low molecular weight (e.g. gallium) hence they will be easily degraded and eliminated by the renal infiltration system. Dendrimer-conjugated contrasting agents display prolonged blood circulation time [52]. A multifunctional PAMAM dendrimer was used as a template to encapsulate gadolinium oxide nanoparticles (Gd_2_O_3_ NPs) for enhancement in vivo magnetic resonance imaging [53]. The PAMAM-Gd_2_O_3_ nanoparticles exhibited a longer longitudinal relaxation time (T1) and better biocompatibility than the clinically popular Gd-DTPA contrasting agents. PAMAM dendrimers coated with magnetite nanoparticles (Fe_3_O_4_) are reported as successful nanoplatforms for combined therapeutic and diagnostic purposes with excellent contrasting properties in MRI [54]. Design and development of such multifunctional model systems has significant potential in anticancer therapy.

### Hydrogel

**Fig. 3:**
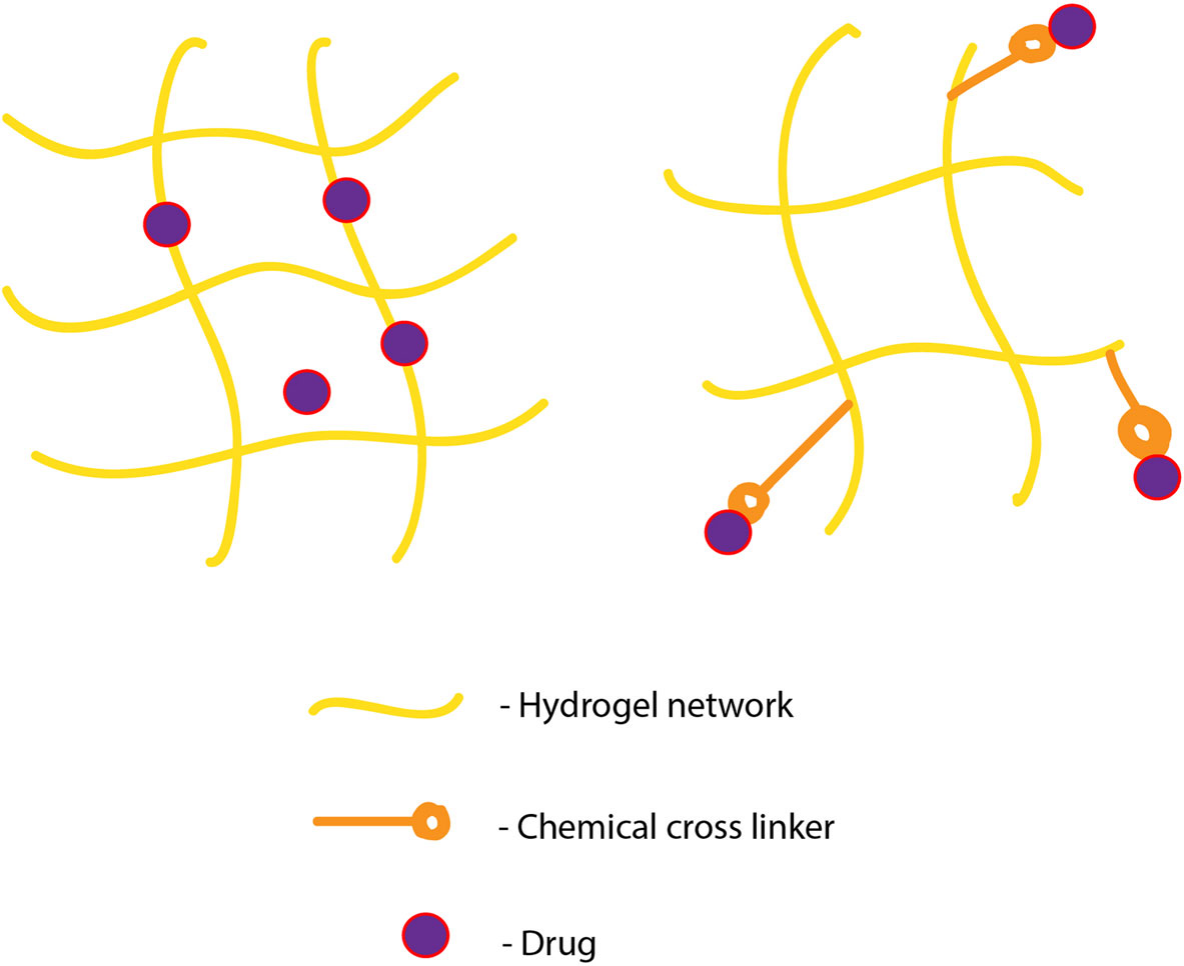
Formulation of hydrogel-drug matrix. The most routinely followed strategy for drug delivery from the hydrogel matrix is physical or chemical interactions. In physical interactions, the affinity between the gel and drug is often charge based. If the gel matrix is having more amino functional groups it could be useful for the delayed release of anionic drugs. Simply, the polymers can have significant effect on prolonged release of drugs of opposite charge. As opposed to physical interaction, drug is permanently linked to hydrogel matrix via covalent crosslinks. This kind of binding could be achieved with other methods like UV irradiation and redox-responsive supramolecular assembly

Hydrogels are three-dimensional polymeric networks and contain greater than 90% of water because of its hydrophilic nature. Biopolymers like chitosan and hyaluronic acid (HA) are the top-line macromolecules used for cancer therapy and imaging. Hydrogel fabrication techniques and usage are increasingly common for pharmaceutical and biomedical applications (Fig. 3) [55–59]. Chitosan-based hydrogels are in absolute demand for drug-delivery applications. Intelligent hydrogels are classified under smart biomaterials because the sensitivity and application of such hydrogels are regulated by external stimulus of temperature, pH, photo and magnetic factors [60]. Photosensitive azidehydroxyethyl chitosan (AZ-HECTS) synthesized by UV radiation has shown biodegradable and biocompatible property with sustained heparin release [61]. A redox-responsive supramolecular hydrogel is a kind of smart or intelligent hydrogel that has been described for the successful delivery of 10-hydroxy camptothecin (HCPT) peptide as a potential anticancer agent [62]. Temperature-sensitive hydrogel like poly(N-isopropyl acrylamide), pNIPA, has significant interest in drug delivery and it exhibits volume phase transition at 32 °C. Below this temperature, water soluble drugs can be encapsulated and the amide groups initiated hydrogen bonds in pNIPA hydrogels are cleaved above 32 °C, resulting in controlled drug release [63]. pH sensitive hydroxyethylacryl chitosan (HC) and sodium alginate (SA) hydrogel were reported for the drug paracetamol under in vitro conditions [64].

#### Nano hydrogel

It is known that chronic inflammation is strongly tied to the initiation and progression of cancer. Hence, an anionic polysaccharide gellan gum based nanohydrogel was developed to offer the dual benefits of anti-inflammatory and anticancer features by chemically cross-linking glucocorticosteroid prednisolone and physically encapsulating paclitaxel [65]. The nanohydrogel system has offered synergistic drug effect from the incorporated drugs by facilitating solubility, drug-uptake and targeted tumorigenesis inhibition via attacking inflammatory components and malignant cells. Systemic chemotherapy is still a preferred first line treatment for solid tumors as it offers effective therapeutic drug load to cancer cells, prolonging drug activity and decreasing the side effects to normal cells. 5-Fluorouracil (5-FU) has been classified as an anti-metabolite with anti-neoplastic activity but having the disadvantage of poor half-life (16 min) and being rapidly metabolized by dihydropyrimidine dehydrogenase. The drawbacks of direct administration of 5-FU is proposed to be greatly reverted by the thermo-sensitive methylcellulose nanohydrogel containing 5-FU and it could be used as an effective systemic chemotherapy for solid tumors such as head and neck cancers, colorectal cancer and brain tumor [66].

### Inorganic nanoparticles

Inorganic nanoparticles exhibit different material properties and hence have many potential applications. The optical and magnetic properties of inorganic nanoparticles have paved way into their usage in cancer therapies. They also exhibit features such as fluorescence, near-infrared (NIR) absorption and Raman enhancement making them extremely useful in image-guided therapies. Inorganic nanoparticles derived from their macromolecule counterparts such as iron oxide, gold or silica have emerged as highly valuable building blocks. Owing to their multifunctional properties, inorganic nanoparticles (gold and iron oxide) were found to be suitable in computed tomography (CT), surface plasmon resonance (SPR), magnetic resonance imaging (MRI), or positron emission tomography (PET) as image contrast agent [67]. Accordingly, the scope of inorganic nanoparticle in image-guided early disease screening has vastly improved. Similarly, the term “multi-modal imaging” has become recently popular as it offers two or more imaging techniques to retrieve more information and permit an effective treatment plan. The multifunctional nanoparticle system containing Prussian blue (PB), serum albumin (BSA), and indocyanine green (ICG) was reported as a novel theranostic agent since it could provide dual-mode magnetic resonance (MR) and near-infrared (NIR) fluorescence imaging in photothermal and photodynamic (PTT-PDT) therapy [68]. Gold (Au) capped magnetic core/mesoporous silica shell nanoparticles were fabricated to obtain the synergistic effect of combined photothermal/chemo-therapy and multimodal imaging in a single system [69]. Nanoparticles made of Au or Ag conjugated with polyethyleneimine (PEI) have also been used to deliver genes [70]. Functionalization of Au NPs with PEG and coumarin were found to efficient incorporation capacity into breast cancer cells without any observed toxicity to other normal cells. A major limitation of using inorganic nanoparticles is that their long-term toxicity and clearance have not been evaluated sufficiently [71].

#### Magnetic nanoparticles (MNPs)

**Fig. 4:**
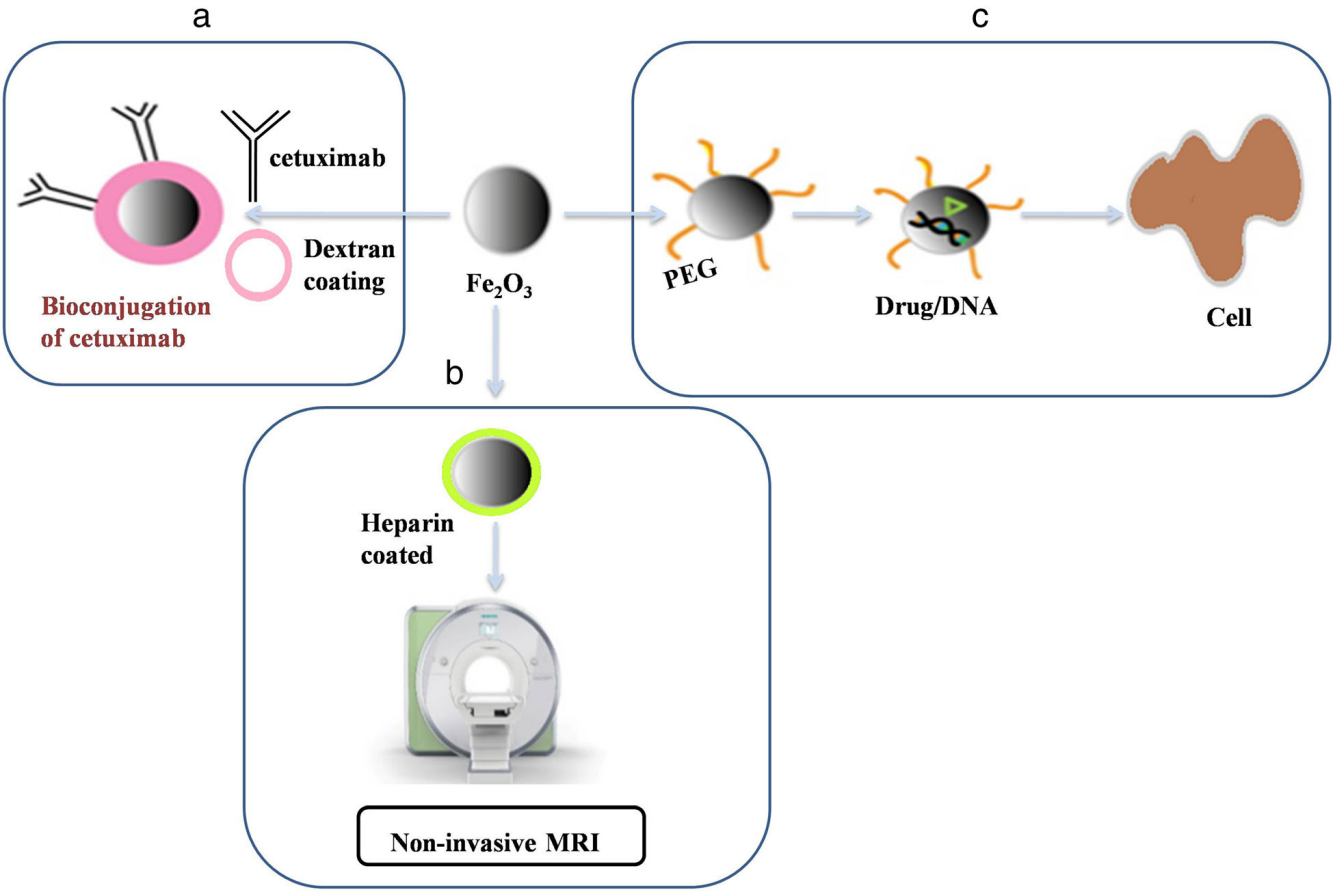
Versatility of magnetic nanoparticles in biomedicine. **a** Iron oxide nanoparticles coated with dextran were subsequently exposed to dihydrazide-PEG linker. This magnetic nanocarrier is useful for bioconjugation of aldehyde bearing cetuximab. **b** Heparin coated super paramagnetic iron oxide nanoparticles are applied for non-invasive MRI **c** A suitable polymer coated spions are successful in delivering any molecules (Drug or DNA) with therapeutic effects

MNPs are distinctively different from other typical nanoparticles due to their unique magnetic property. The main limitations of MNPs are burst drug release and low stability features. To overcome this issue, surface ligands are attached to MNPs, which in turn improve the stability and solubility in biological environments along with exhibiting lesser side effects [72]. Owing to the MNPs large-surface-to-volume ratio, it offers numerous chemically active-sites for biomolecule conjugation (Fig. 4). Thus, it provides longer circulation time, target-specific binding and drug delivery [73]. As of now, chemotherapy, radiotherapy, and medical procedures are considered the three clinically accessible treatments in tumor management. The main drawbacks of these treatments are the side effects as they are not specific. As an alternative to this, thermotherapy is being used to kill a tumor cell with principles based on the higher themo-sensitive nature of cancer cells than normal cells. This can be achieved by hyperthermia in which the temperature of a local region or the body is increased up to 40–45 °C through radiation. The second method, thermo-ablation, uses above 45 °C temperatures to the diseased area to destroy tissues. In animal models, MNP-mediated hyperthermia has been successfully used for the treatment of mice tumors [74]. Their magnetic property is not only useful in magnetic separation and magnetic resonance imaging but also useful in many applications; namely tissue engineering, gene transfection, magnetic memory devices, and magnetic ink, etc. The application of MNPs can also be extended to drug targeting and cell sorting [75]. Paclitaxel (PTX) or rapamycin loaded glycerol mono-oleate-coated magnetic nanoparticles (GMO-MNPs) conjugated human epidermal growth factor receptor 2(HER2) antibody showed 24 times more effective anticancer activity than the free drug [76]. The potential for nanotoxicity exists and presents a great concern; yet research in this fascinating area continues.

#### Carbon nanotubes

Carbon nanotubes (CNTs) are defined by the hexagonal arrangement of carbon atoms that leads to cylindrical nanostructure formation. Arc discharge, laser ablation, and chemical vapor deposition are some of the imperative methods for the production of CNTs. Graphene sheets are rolled at certain angles to create desired CNTs and the said nanotubes are either classified as single wall (SWCNT) or multi-wall (MWCNT) depending on the layer of graphene sheets. The outer diameter of SWCNT is typically between 0.4 and 2.0 nm and between 10 and 100 nm for MWCNTs [77]. CNTs have unique electrical, mechanical and optical properties along with a high surface area that make them appropriate for attaching biological cargoes. Originally, CNTs were toxic because of their hydrophobic surface and limited aqueous solubility. As a result, CNT mediated the following harmful effects by free radical formation, reactive oxygen species (ROS), apoptosis, granuloma formation, and increased inflammatory responses. This toxicity of CNTs can be overcome by proper functionalization methods. The structural feature of CNT is better utilized for changing the surface of the CNT, i.e., the inner hollow structure is used to accommodate suitable drugs and the outer surface is modified via physical or chemical bonding [78]. The CNT surface can be customized with molecules of choice by adsorption, electrostatic interaction or covalent bonding that render them hydrophilic [79].

**Fig. 5:**
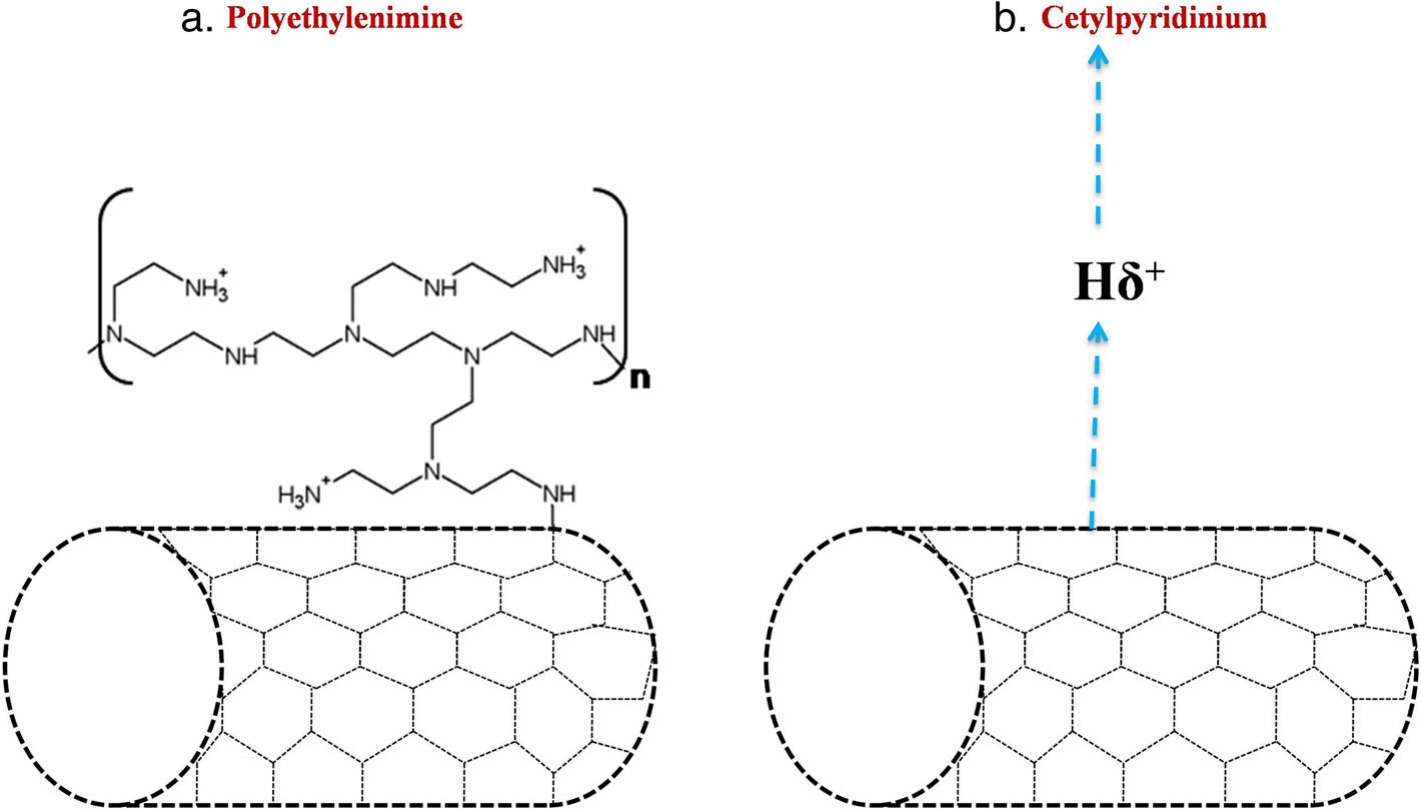
CNT functionalization for siRNA delivery. To achieve an effective siRNA delivery, CNTs were functionalized with covalent and non-covalent crosslinking. **a** CNT covalently linked with cationic polymer polyethylenimine (PEI) **b** CNT functionalized with non-covalent interaction with cationic cetylpyridinium. The different functionalization methods were tried to achieve efficient gene silencing. A thin and long structural feature of CNT offers long surface area and nano-needle morphology facilitates easy translocation over the plasma membrane via endocytosis-independent pathway

Multi-functionalization strategy is an interesting concept wherein CNT can be functionalized with a fluorescent probe and amphotericin B to examine cellular uptake and controlled drug delivery. Surface engineered CNTs are taken up into cells by endocytosis, phagocytosis or membrane translocation; however, certain properties like tube dimensions, surface functionalization and the cell type determine the uptake rate [80]. Higher drug loading on the surface or inner core, ease of conjugation with ligands, thermal ablation and easy cellular uptake are attractive CNT features in cancer treatment and diagnostics [81]. They can target deliver anticancer drugs to arrest cancer cells progression. CNTs have also been used to carry topoisomerase I inhibitors (topotecan) and topoisomerase II inhibitors (teniposide) to slow the growth of cancer cells down by inhibiting DNA topoisomerase activity [82]. Similar to nanomaterials drug delivery, CNTs have been used in transfection for delivering genes or DNA to mammalian cells. Recently, siRNA based therapy was found to be attractive for the treatment of various diseases including cancer. However, siRNAs are prone to easy degradation by RNases, henceforth effective strategies are requisite for delivering the siRNA molecules. Non-covalently functionalized SWCNTs by PEI conjugated to 1,2-distearoyl-sn-glycero-3-phosphoethanolamine-N-[amino (polyethylene glycol)-2000] (DSPE-PEG-PEI) were successful in facilitating siRNA delivery in vitro as well as in vivo (Fig. 5) [83]. CNTs were also used in neuron-repair strategies or neuro-tissue engineering as nerve tissue reconstructing platforms [84]. They could act as an electrical interface for neuronal stimulation, recording [85] both in vitro and in vivo, and also promote neuronal survival, differentiation, growth, and performance [86].

#### Quantum dots

**Fig. 6:**
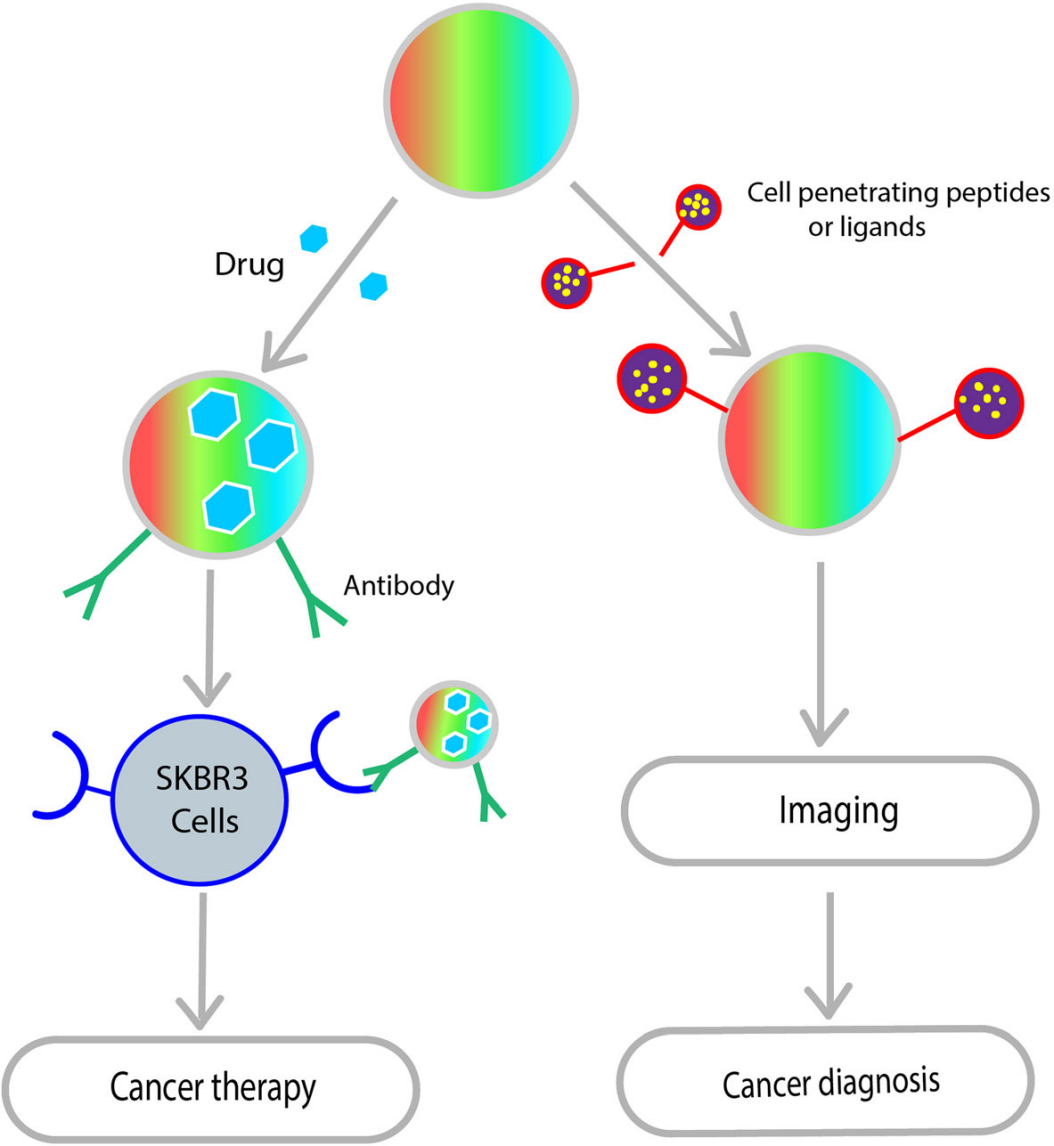
Ambidextrous nature of QDs in nanomedicine. Theranostics is particularly useful to establish specific or molecular targeting in a single agent (QDs). A range of fluorescent semi-conducting nanocrystals can acts as theranostic agent. Because of its ability to accommodate various functional modalities either targeting agents (antibody, aptamer or protein) or cell-penetrating ligands can be incorporated into QDs for cancer therapy or diagnosis

Quantum dots (QDs) are nanosized semiconductor particles (2–10 nm) prepared from chalcogenides (selenides or sulfides) of cadmium or zinc. In general, the size and shape of the quantum dots will determine its optoelectronic properties. Longer quantum dots (radius of 5–6 nm) will emit orange or red color and the smaller QDs (radius of 2–3 nm) emit the colors blue and green [87]. From an application perspective, QDs are prepared like core-shell structures with an appropriate functional coating through a high-temperature strategy which yielded particle size of < 10 nm with narrow size distribution [17]. The versatile surface chemistry and photo-physical property allow the preparation of multifunctional QDs for drug loading, targeting, controlled release, and monitoring of pharmacokinetics and biodistribution [74]. Multifunctional nanocomposite, i.e., carboxyl modified, QDs are crosslinked with amino-functionalized immune-liposomes. These are prepared with anti-human epidermal growth factor receptor 2 (anti-HER2) scFv for cancer diagnostics and targeted therapeutics in HER2 overexpressing human breast carcinoma cells, SK-BR-3 and MCF7-C18 (Fig. 6) [88]. With further technological advances, fluorescent carbon quantum dots (CQDs) have emerged as a potential entrant to traditional semiconductor quantum dots. As quantum dots, CQDs have been used in sensing, imaging and medicinal applications [89].

## Physical properties of nanoparticle

### Optical property

Noble metal nanoparticles (Cu, Ag, and Au) are known for their unique optical properties exhibited near UV and visible spectral wavelength range. Furthermore, the optical properties of such nanoparticles are used to attain desired contrast in various cell imaging applications. Gold nanoparticles (AuNPs) are extensively used in optical imaging due to their unique plasmonic properties, hence AuNPs-assisted bioimaging is mainly used in direct visualization, monitoring of biomolecular events and physiological process and in vivo deep-tissue imaging [90]. The modification of size and shape of the gold nanoparticles covered with borosilicate glasses have induced greater variations in optical properties [91]. Metallic nanoparticles like Ag nanoparticles (Ag NPs) can exhibit controllable optical properties and the optical property could be enhanced when it is combined with organic solar cells (OSC). Reactive oxygen species or ROS are a group of highly reactive molecules involved in many cellular processes, especially chronic diseases like cancer, diabetes and heart diseases. Additionally, they play an important role in cell signaling pathways; hence, quantification of ROS is highly sought. Traditional optical-based analytical techniques have suffered from low-detection limit fluorescent-gold (Au) nanoparticles having the potential alternative to improve sensitivity. More advanced methods are often introduced to overcome the aforementioned limitations. In this regard, an enzyme immobilized onto graphene-electrode was developed for biosensors applications. A single nanoparticle (Au-Pt) based optical sensor was investigated at a nanomolar level for the detection of ROS from microbes and aquatic environments [92].

UV light-responsive drug delivery system (DDS) has its own disadvantages including poor tissue penetration and cell toxicity as a result of UV light exposure and hence is not suitable for clinical practice. Therefore, NIR is considered more promising for clinical application due to spatiotemporal control and considerable penetration [93]. As part of anticancer therapy, multifunctional dimercaptosuccinic acid (DMSA) coated iron oxide (Fe_3_O_4_) nanoparticles with doxorubicin (DOX) have demonstrated excellent cell toxicity to human breast cancer (MDA-MB-231) cells via the synergistic effect of pH and NIR-light induced photothermal therapy combined with chemotherapy [94]. Based on these results, it was stated that the Fe_3_O_4_@DMSA/DOX nanoparticles may work as an effective anticancer therapy for breast cancer. With suitable surface modification, a multifunctional Zn-Fe_2_O_4_ nanoparticle was designed as an anticancer drug carrier for the hydrophobic molecule curcumin and the hydrophilic molecule daunorubicin in cancer therapy [95]. The hydrophobic-hydrophobic interaction between curcumin and long-chain surface ligands of Zn-Fe_2_O_4_ nanoparticles favored the incorporation of the drug molecules into the alkyl chain of oleic acid-coated Zn-Fe_2_O_4_ nanoparticles. Whereas, the daunorubicin drug molecules were adsorbed on the surface of the nanoparticles via electrostatic interaction. Development of such multifunctional nanoparticles has been found to have promising application in dual drug delivery applications.

### Magnetic property

The concept of magnetic property in drug delivery was introduced in the year 1978 [96]. Considering the technical advancements in MNPs design and in vivo studies, MNP based drug delivery has received much attention in the field of nanomedicine [97]. The route of administration will have a direct impact on poor drug bioavailability, especially if administrated via the systemic route due to incomplete absorption or degradation. In conventional drug delivery (i.e., injection or ingestion of the desired drug), each drug has its own therapeutic range above which it is toxic and below which it is ineffective. This is because the oscillating drug concentration will result in either ineffectiveness or toxicity [98]. To overcome the limitations of conventional drug delivery system, specific target-hitting drug delivery systems are required and anticipated to provide more effective drug accumulation in the diseased site. Even in targeted drug delivery, a significant quantity of injected PEGylated liposomal DOX was seized by lysosomal sequestering after an internalization that resulted in limited efficacy of the drug [99].

A magnetic drug delivery system is generally comprised of an iron oxide nanoparticle with the drug of interest and they are delivered to the tumor site with the aid of external magnetic field [100]. The magnetic property is not only useful in delivering drugs to the target site but also useful in gene delivery, a contrasting agent in MRI and cell separation, etc. Among the various MNPs, iron oxide is the most preferred material as it is biocompatible, biodegradable and most importantly, possesses superparamagnetic (SPM) behavior [101]. The MNPs are often coated with polymers such as dextran, starch, and PEG to stabilize the core iron oxide nanoparticles. Consequently, particle aggregation will be reduced; however, it also decreases the magnetization saturation of bare iron oxide nanoparticles. The SPM behavior of nanoparticles is defined by lack of hysteresis loop, coercivity and remanent magnetization at room temperature [102]. When the nanoparticles are synthesized at the size range of 10 nm without surface modification, it offers negligible remanence and coercivity in the magnetization curves. The small size and surface effect of the particle will determine the magnetic responsiveness of the material [103, 104].

Designing of such nano-scale SPM materials has a significant impact on nanomedicine including magnetic resonance imaging application for neuro-oncology [105], drug delivery via magnetic drug targeting [106] and enhanced hyperthermia by iron oxide nanorods [107]. The dual targeting of drug delivery by magnetic nanoparticles (MNPs) combined with liposomes is another recent trend in cancer therapy [108]. Bubble-generating magnetic liposomal (BML) drug delivery system is triggered with drug release properties for targeted delivery of doxorubicin in cancer therapy. BML was obtained by treatment of liposomes with citric acid-coated iron oxide MNPs co-entrapped with ammonium bicarbonate by simple hydration and surface modified with hyaluronic acid-polyethylene glycol (HA-PEG) coating [109]. The resultant liposomes are effective in delivering increased DOX concentration to the human glioblastoma cells (U87) cancer cells through temperature-sensitive drug release thereby improving targeting as well as treatment efficiency.

### Particle size

Nanoparticle applications are predominantly governed by its properties wherein particle size and size distribution are crucial as size will easily influence the drug loading, release, toxicity, in vivo distribution and particle stability, etc. One of the biggest limitations in nanoparticle aided drug delivery is clearance by the reticuloendothelial system (RES) through opsonization and it is implicit here that the size influences clearance as well as distribution. When the particle size exceeds 100 nm, the pharmacokinetic and biodistribution properties greatly change and they are detected in blood and organs like spleen, lungs, liver, and kidney [110]. Positively charged NPs show better uptake by direct permeation than neutral and negatively charged NPs [111]. Nanoparticle size or the particle diameter can be controlled either by the fabrication methods or adjusting the physical properties, particularly concentration of the polymer or the surfactant. For brain targeted drug delivery systems, the difficulty of treating brain tumor is overcome by shrinking endothelial cells and opening endothelium tight junctions for the delivery of chemotherapeutics across the blood-brain barrier (BBB) [112]. To improve the paracellular transport, tight junctions can be opened only to a certain extent and particles of < 20 nm can penetrate the brain via the BBB. The BBB disrupting properties of hyper-osmotic mannitol facilitate effective penetration of nanoparticles across the BBB [113]. For such effective penetration, the particle diameter should ideally be 10–150 nm as it will sustain longer circulation time and increased accumulation in the target site [114]. The rate of drug release can be tuned by particle size and, in case of large particles, more drug molecules can be accommodated and slowly released [115]. Although the smaller nanoparticle has a high surface-volume ratio, they can easily be aggregated and may be released quickly since they adhere to the edge of the particle surface.

### Morphology

It is clear that the number of nanoparticle properties i.e., particle size, charge and surface have a great effect on drug delivery. Besides, nanoparticle shape has also been significantly useful in the development of nanocarriers (NC). The significance of nanoparticle shape in drug delivery has been discussed by several authors [116, 117]. However, the precise role of particle shape in drug delivery has yet to be delineated. The shape of the nanoparticle is always dependent on the synthesizing methods where methods like ab initio are used to produce particles with non-spherical geometry [118]. Since the non-spherical particles may have two or more different lengths, one length could dominate the other. Irrespective of the different administration routes, particle shape will greatly affect the transport and diffusion of nanoparticles. It has been shown that the sphere-shaped particles move easily due to their inherent symmetry whereas the non-spherical ones tumble with the flow. This will be more prominent in filtering organs like spleen and liver. Folic acid-targeting folate ligands in the form of spherical and wormlike micelles (75 and 200 nm) using acrylic acid (AA) and PEG methyl-ether acrylate (PEGMEA) were intended for drug delivery [119]. When compared to spherical particles, wormlike micelles were highly accumulated in the spleen, liver, and kidneys. Long filomicelles should be stretched out whenever νflow> 5 μm s^− 1^, which includes flow in most blood vessels and also the filtering spleen [120]. It was reported that the shape, geometry, and orientation of the particle would greatly influence the cellular uptake [121, 122] and, even in cases of non-spherical particles larger than 200 nm, can still pass through the spleen provided one of their dimensions is less than 200 nm [123]. The target-specificity of nanoparticles is also subjected to the shape of the nanoparticle which may eventually result in longevity and internalization of particles. Therefore, it was concluded that the symmetry of nanoparticle is crucial for effective drug delivery.

### Surface tailoring

The wide-spread clinical applications of nanoparticles fostered studying the interaction between the nanoparticle surface and the inner biological system, especially, at physiological conditions (pH of 7.0 to 7.4). Based on the choice of application, a nanoparticle with desired property is selected, e.g., optical-gold NPs [90], magnetic–iron oxide nanoparticle (IONPs) [124, 125], fluorescence-quantum dot, etc. [126]. Before introducing such a nanoparticle into the environment, it needs to be carefully modified with the appropriate functional groups by a suitable fabrication method. The mentioned surface engineering approaches not only offer an excellent stabilization in an aquatic system but also effectively deliver the drug to the target site. The particle stabilization is often achieved through ligand immobilization or polymer coating. Binding of a ligand on the surface of a nanoparticle would prevent agglomeration by a repulsive force, which results in the control of nanoparticle size and shape [127]. When the nanocarriers are introduced into the biological system, the proteins in the biological fluids will commence being adsorbed into the nano surface and form a protein-rich layer (protein corona). The resultant protein-corona and nanoparticle complex have protective effects on the biological system; however, the molecular complexity of protein-corona nanocarrier is still not well investigated [128]. Formation of protein-corona will occupy the surface of nanoparticles and block the chemical functionality as offered by the nanoparticle. Besides, it will have effects on particle size and size distribution, which directly influences the circulation time, intracellular trafficking and clearance/cell uptake process [129]. Likewise, nanoparticle surface chemistry plays a key role in the cellular uptake process. Polymer coating of the NP surface has considerably reduced the chance of particle clearance by the immune system and avoided accumulation in other organs [130]. The benefits of the polymer coating (e.g. PEG) is to control protein or peptide absorption via its hydrophilic chains that will also regulate cell behavior during contact. Desirable functionality can be added to the particle by methods utilizing monotopic capping agents. However, fabricating this the right way still remains a challenge [131].

## Drug-laden nanocarriers

The name “nanocarrier” suggests that the materials belonging to this category are 1–100 nm, but size ranges > 200 nm are generally to be avoided because particle size has a significant effect on circulation time. This is especially true with smallest capillary dimension as the possibility of obstruction exists. Drug-laden nanocarriers are prepared by various synthesis methods and one of the best-suited methods is nano-encapsulation [132]. Emulsion polymerization is a method wherein natural or synthetic polymers are subjected to a continuous aqueous or organic phase. The selection of nanoparticle-drug formulation is decided by the physicochemical properties of the drug viz. drug solubility nature, chemical stability, etc. In the continuous organic phase methodology, polymers are added along with the surfactants to prevent aggregation. The method also exploits initiators and toxic organic solvents for preparation. Hence greater emphasis is placed on alternate methods with more safety. In the continuous aqueous phase, mostly antibiotic or drug molecules are encapsulated in the nanoparticle using aqueous solution without surfactant or emulsifiers. The synthesis of poly (methyl methacrylate) (PMMA) nanoparticle to carry influenza viral adjuvant is a classic example of continuous aqueous phase polymerization produced through radical emulsion polymerization [133]. In addition to the antigen example, various drugs like doxorubicin, ketoprofen, and insulin were also nano-encapsulated [134].

Some of the nanoparticle formulations have offered improved and higher oral availability of low-water soluble drugs. Most of the anticancer drugs (paclitaxel, docetaxel), small molecule anticancer drugs [VEGFR inhibitors (e.g. cabozantinib, nintedanib] and compounds like curcumin have exhibited poor solubility and, even today, the solubility range of recently developed anticancer compounds are at the μg/mL range [135–137]. The feasibility of using a nanocarrier is not restricted to improving the bioavailability. It also has additional benefits: reduced systemic toxicity, enhanced tumor accumulation and improved therapeutic effectiveness by selective drug aggregation [138]. Among the different types of nanocarrier systems, nano-formulations based on lipid, polymer, and albumin are widely studied for its encapsulation and delivery of the existing as well as new chemotherapeutic drugs. Pyrazolo[3,4-d] pyrimidines demonstrated promising anticancer activity against many different cell lines but like many other anticancer compounds, it displayed poor aqueous solubility. This potential limitation was overcome by encapsulating in nanosystems like albumin nanoparticles and liposome and in the end, it showed remarkable pharmacokinetic profile [139].

Although therapeutic proteins are approved by the FDA for various disease-designated purposes, the main drawback of therapeutic proteins is that low half-life and lack of stability. Liu et al prepared interferon conjugated with an alpha block copolymer to form IFN-POEGMA-PHPMA [poly (oligo (ethylene glycol) methyl ether methacrylate)-poly(2-hydroxypropyl methacrylate)] micelle and compared its tumor activity against the US FDA approved IFN-α PEGASYS (Peginterferon α-2a). The results showed complete suppression of tumor in mice model when administered with IFN-micelle. While PEGASYS and IFN-POEGMA were effective, IFN at the same dose (1 mg/kg) was not as efficacious. The in vitro bioactivity of the micelle was 21.5 fold higher than that of the FDA approved interferon. The result indicates that stability and therapeutic efficiency can be increased by conjugating with polymer [140].

### Factors influencing the biodistribution of drug-laden nanocarriers

It is believed that numerous factors could affect drug loading capacity besides synthesis methods and reaction conditions. Chitosan-grafted-glycerides (monooleate, monolaurate, and monostearate) were synthesized to achieve a successful transport of drugs across the complex intestinal barrier. This study also reported that the selection of optimum co-polymer and drug is equally essential in the preparation of a stable micelle system and it was achieved using computational simulation [141]. Each nanoparticle or nanocarrier system has a distinct chemical composition and size variation. If the carrier is not surface modified with suitable agents, it is rapidly cleared from the bloodstream by mononuclear phagocyte system (MPS) (this process is called phagocytosis), the liver or the spleen [142]. Hence, an important aspect of designing nanocarrier is fabricating the nanosystem with optimal clearance characteristics with particle material, size, shape, surface chemistry, and charge being some of the properties that would influence this clearance. Ideally, the size would be bigger than blood capillaries to avoid leakage yet tiny enough to hide away from macrophage engulfment. To overcome the numerous biological barriers, surface modified carriers are increasingly described for targeted drug delivery and it could be achieved by incorporating desired functionality or characteristics on the nanoparticle by suitable synthesis methods. The surface-modified carriers are expected to provide prolonged circulation time and minimize the risk of opsonization. P-glycoprotein (P-gp) is an efflux membrane transporter found to be overexpressed in cancer cells and act as a physiological barrier. It obstructs chemotherapeutic agents from entering the cytosol by extruding them to the exterior during anti-tumor treatments [143]. Polysorbate 80 has been demonstrated as an inhibitor of P-gp and its potential P-gp inhibition results in the delivery of a significant amount of doxorubicin using nanoparticles with polysorbate 80 coating [144].

## Targeted drug delivery methods

### Passive targeting

**Fig. 7:**
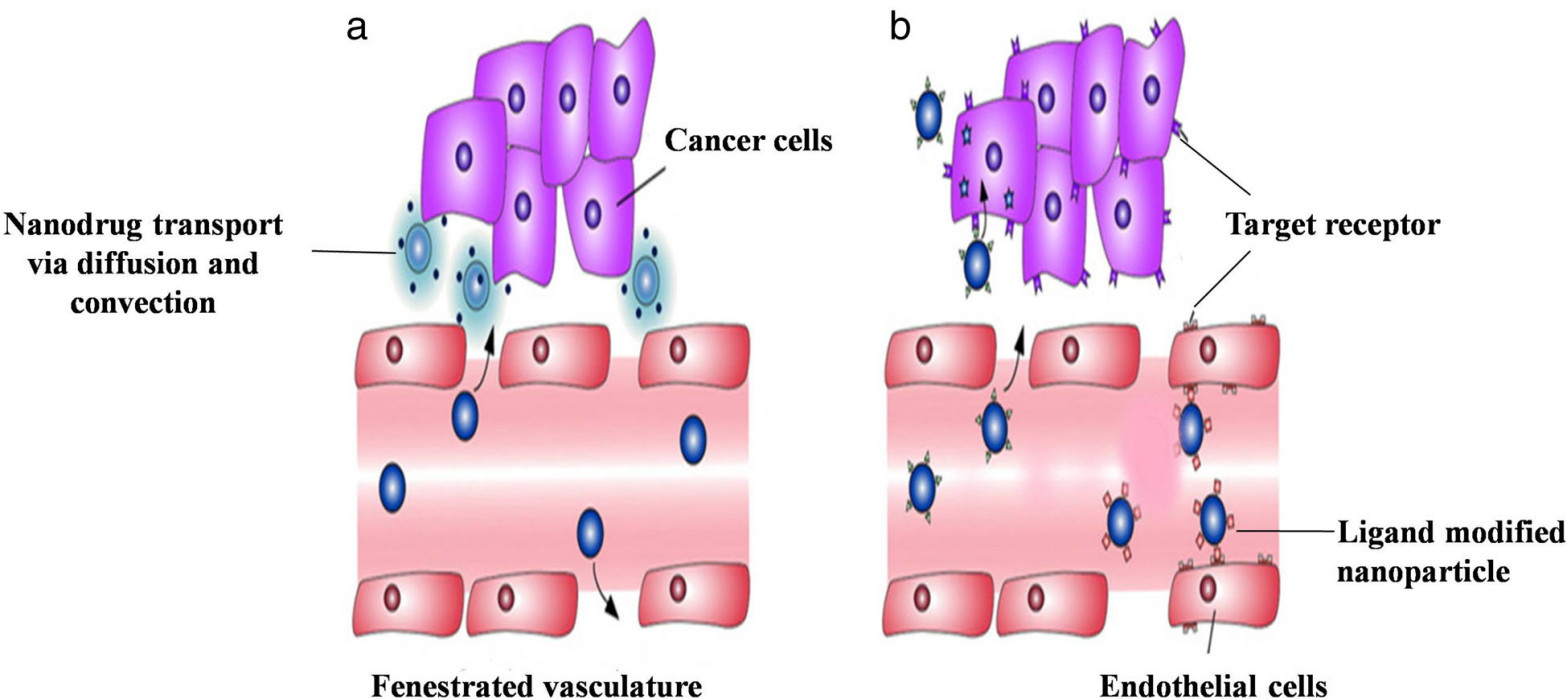
Drug delivery through passive and active targeting. Enhanced vascular permeability is one such hallmark feature of tumor cells along with the defective vascular anatomy. **a** Passive targeting uses this feature and improves the drug delivery by convection or passive diffusion in tumor cells. **b** Whereas in active targeting, targeting ligands are over expressed in tumor cells, thus the coveted nanoparticles are engineered to incorporate ligand that will bind to the target cells through ligand receptor interaction. This in turn increase the efficiency of drug delivery to the tumor tissues [Adapted from reference with permission: Wicki A, Witzigmann D, Balasubramanian V, Huwyler J Nanomedicine in cancer therapy: challenges, opportunities, and clinical applications. J Control Release 2015; 200:138–157] [154]

Drug targeting is defined as the selective drug release at a specific physiological destination organ or tissue or cell in which specific pharmacological impact is required. Nanocarrier mediated cell targeting includes active and passive mechanisms. In passive targeting, the drugs can be delivered to the target organ passively based on the longevity of the pharmaceutical carrier in the blood and preferential accumulation of the drug-loaded nano delivery system at the site of interest [145]. The main property of tumor tissues is that they have defective blood vessels and hence exhibit increased vascular permeability. This unique characteristic helps to transport macromolecules into tumor tissues. Maeda et al have demonstrated that the site of infection or inflammation where excess bradykinin is generated also exhibits enhanced permeability and retention (EPR) effect [146]. The main difference between the infection-induced EPR effect and that of tumor is the duration of the retention period. In the case of normal tissues, the time will be shorter due to swelling while in cancer tissues the lymphatic drainage system is active. Thus swelling may disperse after a few days. In cancer, the enhanced vascular permeability results in adequate nutrients and oxygen supply to the tumor tissues for their rapid growth. This unique anatomical–pathophysiological nature of tumor blood vessels is being exploited to deliver drug molecules to the tumor tissues. Macromolecules bigger than 40 kDa will spill out from the tumor vessel and concentrate in tumor tissues. Normal tissues lack this EPR effect driven drug delivery. This unique EPR effect feature of tumor cells is subsequently thought to be a milestone principle in tumor-targeting chemotherapy and is turning into an inexorably encouraging worldview approach for anticancer drug development. Hence, it has become the golden standard in anticancer drug design and anticancer strategies using macromolecular agents, including gene delivery, molecular imaging, antibody therapy, micelles, liposomes, and protein-polymer conjugates [147–149]. PEG is the most important polymer used to modify proteins to enhance the efficiency of drug delivery. PEGylated L-asparaginase has a circulation lifetime of 5.7 days in humans compared to 1.2 days for the original enzyme [6] and was successfully used as induction therapy for phase-3 acute lymphoblastic leukemia (ALL) [150]. Several proteins–polymer conjugates are already available as anticancer agents. In some cases, blood plasma components are capable of increasing circulation time. A study by Gradishar et al revealed higher response when nanometer-sized albumin-bound paclitaxel (ABI007) was administered intravenously in women with metastatic breast cancer than standard paclitaxel formulation [151]. Similarly, the ABI007 nano-drug showed a 4.5-fold increase in paclitaxel transport across endothelial cells compared to standard paclitaxel [152]. Taxol®, when loaded into micelles made of PEG-β-poly(4-phenyl-1-butanoate)-l aspartamide conjugate, showed almost a 100-fold increase in the area under the curve (AUC), a 15-fold decrease in the volume of distribution and a significant decrease of drug clearance was achieved resulting in a 25-fold improvement in drug accumulation in C-26 tumors in mice [6]. Polymer-conjugates styrene-maleic anhydride-neocarzinostatin (SMANCS), the PEG-granulocyte colony-stimulating factor and PEG-L-asparaginase are currently available in the market and are being used against hepatocellular carcinoma, acute lymphoblastic leukemia and chemotherapy-associated neutropenia, respectively [153]. Passive targeting cannot deliver large solutes and there arises the need for alternative tactics which has led to the development of active methods (Fig. 7a) [155].

### Active targeting

Active targeting is based on the attachment of a specific site to the surface of pharmaceutical carriers. It makes use of molecular recognition patterns like ligand-receptor, antigen-antibody to deliver drugs to a specific location (Fig. 7b). This strong interaction confers more specificity to the delivery system. The active strategy can be also achieved through the manipulation of physical stimuli (e.g., temperature, pH, magnetism) [138]. In active targeting, the ligand is coupled onto the nanoparticle surface that will interact with its receptor in the target site. The success of drug targeting relies upon the choice of targeting moiety which ought to be abundant, have strong affinity and specificity to bind cell receptors as well as be suitable to chemical modification by conjugation. The active targeting ligands for tumor therapy include folate, transferrin, aptamers, short oligonucleotides of RNA or DNA that can fold into various conformations and engage in ligand binding, antibodies, and peptides, etc. Active targeting offers less toxicity to healthy tissues as targeting ligands are overexpressed on the tumor tissue, so it is widely used for cancer treatment [99, 114, 156]. Poor tumor targetability and multidrug resistance (MDR) are two major impediments to the success of cancer treatments. In the case of specific-drug targeting, internalization of nanoparticles over receptor-mediated cell interactions are considered an effective method. A large number of epithelial cancers have the characteristic overexpression of folate-receptors; hence, they are targeted for effective chemotherapy [157]. Ethoxy-(poly(ethylene glycol))-folic acid (FA-PEG) micelle consist of docetaxel (DTX) used to exert higher toxicity on FR-positive MCF-7 cells [158]. Hyaluronic acid (HA) or its derivatives are increasingly used to target and bind to overexpressed cell-surface receptors on the tumor cells and can deliver various anti-tumor drugs, proteins and nucleic acids [159]. HA-paclitaxel conjugate (HA-PTX) has shown superior anti-tumor activity against head and neck squamous cell carcinoma cell lines OSC-19 and HN5 upon binding to CD44 receptor, increasing the uptake of the polymer-drug conjugate [160].

Fabrication of matrix metalloproteinases (MMP)-responsive smart drug delivery system is a new way to inhibit MMPs expression as MMPs are widely considered cancer biomarkers. Such targeting systems are developed by incorporating the MMP substrates (collagen, gelatin, fibrinogen, etc.) into nanoparticles. However, large proteins have serious limitations in drug targeting or delivery. The synthetic MMP substrates (i.e., MMP-sensitive peptides) are not only easy to incorporate but also offer selectivity and sensitivity. Yet the MMP responsiveness of the nanoparticles varies with the choice of peptides used [161]. A new type of self-assembling polyethylene glycol-phosphoethanolamine-based copolymers (PEG-pp-PE) was designed for treating drug-resistant cancers by inhibiting both the matrix metalloproteinase 2 (MMP2)-sensitive tumor-targeting and P-glycoprotein (P-gp)-mediated drug efflux [162]. The molecule size and surface attributes of nanoparticles can be effectively controlled to accomplish both passive and active drug targeting with fewer side effects. Nanoparticle addition shields the drug from degradation. This system can be utilized for different routes of administration including oral, nasal, parenteral, etc. The drug will remain at a specific site in the right proportion for a prolonged time with less wastage and efficacy [163, 164].

## Different administration routes of Nanocarriers

### Transdermal drug delivery (TDD)

**Fig. 8:**
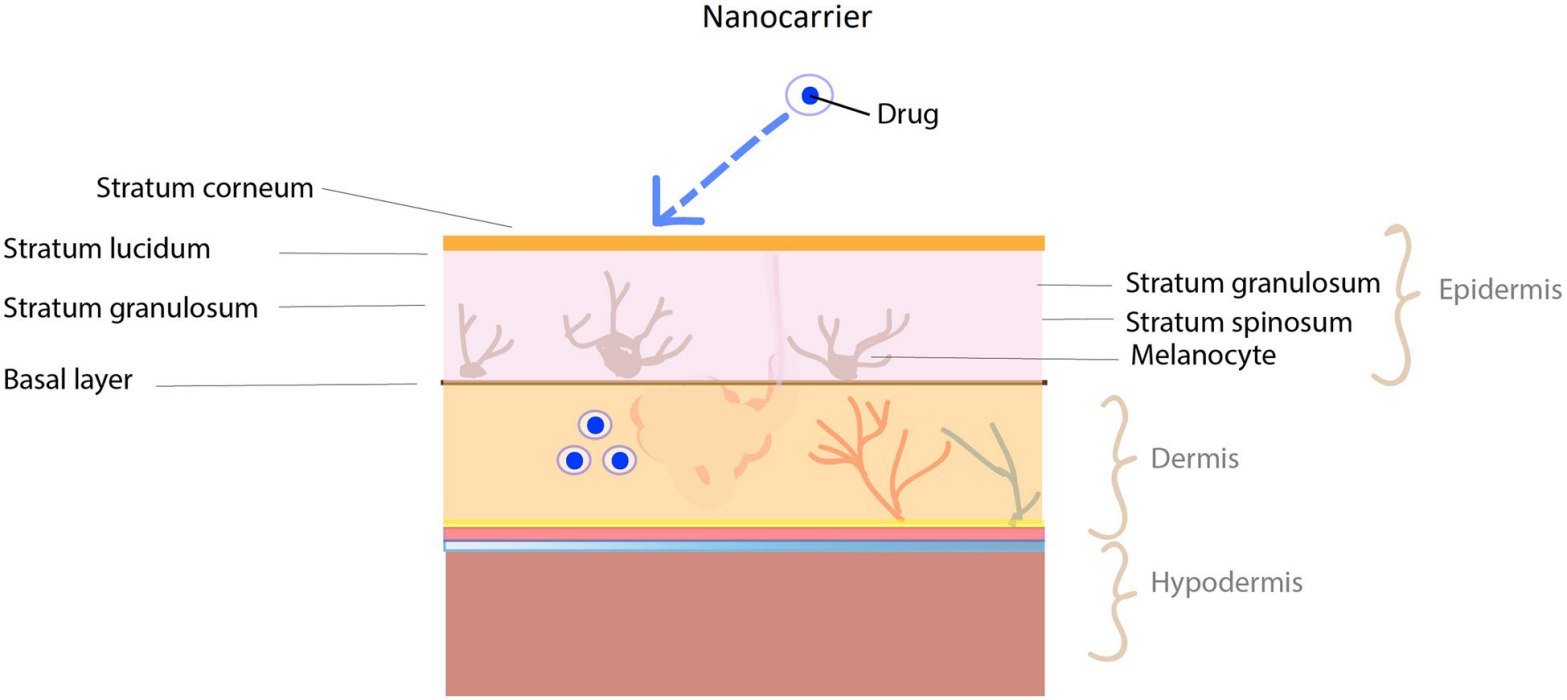
Nanocarrier assisted transdermal drug delivery. Dermal application of the drugs is still promising approach irrespective of the principle skin layers epidermis, dermis and hypodermis which is acting as a barrier and protecting the body. The outer skin layer or the visible “epidermis” further has three distinguished separate layers which limit the penetration of drugs into deeper skin layers. Fabrication of engineered drug laden nanocarriers is designed to overcome the skin barriers and reach the deeper skin layers. The nanocarriers penetration into skin via different pathways is clearly documented and the development of active and passive delivery methods enables the enhanced transdermal delivery

Human skin is the largest organ in our body covering a surface area of 1.8–2.0 m^2^. It is composed of three main layers: the epidermis, dermis, and hypodermis (Fig. 8). The outermost epidermis layer is made up of 95% keratinocytes and the remaining percentage consists of Langerhans cells, melanocytes, and Merkel cells. The outermost layer of the epidermis, stratum corneum consists of anucleated physically dead keratinocytes called corneocytes presenting a thickness of 10–20 μm [165, 166]. The multilayered brick and mortar structure of keratinocytes, together with their lipophilic nature of the stratum corneum is responsible for the barrier property of the skin [167]. The primary goal of a nanocarrier is to overcome the stratum corneum barrier. NCs such as nanoemulsions, vesicular (liposomes, ethosomes, niosomes, etc.) and nanoparticular NCs are developed to overcome this obstruction [168]. Nanoparticles enter the skin through (1) the intercellular pathway (lipid matrix occupying the intercellular spaces of the keratinocytes), (2) the transcellular pathway (through keratinocytes) and (3) the transappendageal pathway (across hair follicles, sebaceous glands, and sweat glands) [169].

Since skin appendages cover only 0.1% of the skin surface area, initially it was considered as a non-important route for drug penetration. Nevertheless studies have shown that hair follicles could be an interesting option for drug penetration through the skin [34]. Lademann et al investigated the storage behavior of the dye containing nanoparticle (320nm) and non-particle form on human skin and found that the nanoparticle formulation stored in hair follicles up to 10 days, while non-particle form could be detected up to 4 days [170]. The surface images of topical administrated polystyrene nanoparticle on porcine skin (ex vivo) and human skin (in vivo) have revealed NPs accumulation in the follicular openings. The CLSM (confocal laser scanning microscopy) images showed accumulation of F-NP (20 nm) were almost the same in hair follicles and skin appendages after 30 minutes. Increasing exposure time for about 1 to 2 hours displayed a better accumulation in hair follicles than skin appendages. The results conveyed the time dependent distribution of naoparticle accumulation in hair follicles [171]. The TDD system prevents the first pass metabolism effect of drugs. Therefore, lower amount of drug can be administered efficiently with reduced toxicity. The main disadvantages of TDD system are that not all drugs can be delivered transdermally. High molecular weight drugs (>500 Da) are not capable of penetrating the stratum corneum [34, 172].

#### Nanocarriers for transdermal drug delivery

The commonly used nanocarriers for dermal/transdermal drug delivery in the pharmaceutical field include liposomes, transfersomes, ethosomes, niosomes, dendrimers, polymer nanoparticles, and nanoemulsions. Liposomes are closed colloidal carriers composed of phospholipids and steroids. They can carry hydrophilic drugs inside their core and lipophilic drugs between the lipid bilayer. Liposomes may be negatively or positively charged and their deformability decreases by increasing the amount of cholesterol in their composition [173]. In 1995, the US FDA approved the first liposome-encapsulated drug Doxil (PEGylated liposome-encapsulated doxorubicin) for the treatment of AIDS-related Kaposi’s sarcoma, later approved for ovarian cancer. Recently, it has also been approved for the treatment of breast cancer in the USA and the treatment of multiple myeloma in combination with Velcade, a proteasome inhibitor, in Europe and Canada [174–176].

To improve skin permeation and increase efficiency, the composition of liposomes are altered to the newly generated classes of lipid vesicles called transferosomes, niosomes, ethosomes, etc. Transfersomes are negatively charged elastic or deformable vesicles composed of phospholipids as their main ingredient with 10 to 25% surfactant (such as sodium cholate) and 3 to 10% ethanol. The presence of surfactants destabilize the lipid bilayers of vesicles and confer their ultradeformability thereby enabling them to squeeze themselves through the narrow pores in the stratum corneum that are less than one-tenth the diameter of the transfersome. While liposomes cannot penetrate the channels, transferosomes, up to 500 nm in size, can penetrate the stratum corneum [177]. Niosomes are vesicular nanocarriers formed by the assembly of non-ionic surfactant in an aqueous phase. Niosomes are developed to deliver both lipophilic and hydrophilic drugs [178]. The unique property of niosomes is that they reduce the systemic absorption of drug which in turn enhances the residence time of the drugs in stratum corneum. The role of surfactant is to enhance the penetration of the drug by adsorption at the interfaces or by interacting with biological membranes and by modifying the barrier function of the stratum corneum. Examples of transdermal drug delivered using niosomes are Minoxidil, an antihypertensive vasodilator medication, and ellagic acid (EA) [34, 179]. Pomegranate ellagic acid (PEA) is a natural polyphenol that possesses excellent antioxidant, anti-tumor, anti-inflammatory, antibacterial, and skin whitening properties. However, the characteristics of low permeability and poor absorption rate of EA have limited its application. The pomegranate ellagic acid-hydroxypropyl-β-cyclodextrin (PEA-HP-β-CD) inclusion complex was prepared to offer an enhanced drug effect via effective transdermal permeation [180]. Similarly, EA-loaded niosomes were also used as an effective carrier for the dermal delivery of EA [181]. Ethosomes consists of the stratum corneum. Ethosomes consist of phospholipid/surfactant, water and ethanol (∼30%). Drugs encapsulated in ethosomes can penetrate deep skin. The presence of a high amounts of ethanol helps in breaking the stratum corneum [182]. Tacrolimus, Clotrimazole, Trihexyphenidyl HCl, Ketoprofen and testosterone have been delivered using ethosomes [34]. Nanoemulsions are a dispersion of oil and water stabilized by an emulsifying agent. Their size varies from 100 to 1000 nm. They are transparent due to the droplet size being less than 25% of the wavelength of visible light [183, 184].

#### Methods involved in transdermal drug delivery

Passive methods can deliver only a limited amount of drug that is of low molecular weight (<500 Da). Active methods have been developed in order to overcome this. It uses mechanical and physical methods to enhance skin permeability. The main advantage of the active strategy is that it can deliver large molecular weight molecules (> 500 Da) efficiently. The novel transdermal delivery system focuses on how to overcome the stratum corneum barrier by using microneedles, thermal ablation, microdermabrasion, high pressure-jets, iontophoresis, laser, electroporation, and ultrasound [174]. Most of these methods are currently progressing to deliver macromolecules (heparin, oligosaccharides) and vaccines. Smallpox vaccine administered by microneedle mediated skin electroporation in mice showed a strong immunological response against the pox virus immunogens of interest than traditional live virus administration [185]. A simple inexpensive microporation has been developed to increase the permeability of the skin for the delivery of genetic vaccine using replication-defective adenoviruses (rdAds) [186]. The resultant skin immunization through microporation is not only painless but also enhances the activity of rdAds by up to 100-fold as compared to intact skin. The advent of these novel strategies has had a great impact on medicine. By addressing the safety, efficacy, portability, user-friendliness, and cost-effectiveness, these novel drug delivery techniques can compete with those already on the market [155, 187–189].

### Blood brain barrier drug delivery

**Fig. 9:**
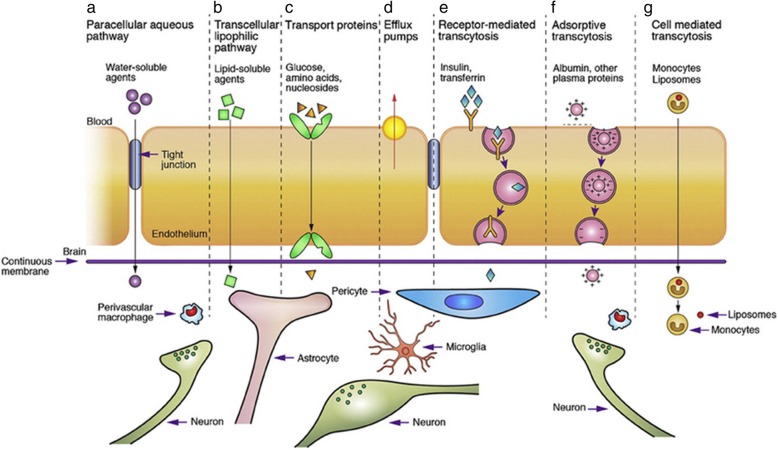
Transport mechanism through blood brain barrier. Transport routes across the blood–brain barrier. Pathways (**a**-**f**) are commonly for solute molecules; and the route (**g**) involves monocytes, macrophages and other immune cells and can be used for any drugs or drugs incorporated liposomes or nanoparticles. [Adapted from reference with permission; N.J. Abbott, L. Ronnback, E. Hansson, Astrocyte-endothelial interactions at the blood–brain barrier, Nat Rev. Neurosci 7 (2006) 41–53] [192]

One of the tightest endothelium, the Blood-Brain Barrier (BBB), is present in the central nervous system. The term “blood-brain barrier” (“Blut-Hirn-Schranke”) was coined by Lewandowsky in 1900. The human brain contains about 100 billion capillaries with the brain capillary endothelium spread across approximately 650 km that covers a total surface area of approximately 20 m^2^. Endothelial cell, astrocyte, pericyte, microglial cells, and adjacent neurons constitute the BBB [190]. The entry of molecules into the brain is regulated by BBB and blood-cerebrospinal fluid barrier. The BBB is considered the bottleneck for successful development of the central nervous system (CNS) acting drugs. Most of the neurotherapeutic compounds never reach the market due to their inability to cross the BBB [191].The high selectivity of the BBB is due to the presence of cerebral endothelial cells. Adherens junctions (AJs) and tight junctions (TJs) present between endothelial cell acts as the physical barrier. This compact network of interconnections supplies transelectrical resistance > 1500 Ωcm^2^ to BBB. Microglia, perivascular macrophages, and mast cell serve as the immunological barrier. The transport barrier includes para- and transcellular routes. The transcellular route includes carrier-mediated transport, receptor-mediated transcytosis, adsorptive mediated transcytosis and cell-mediated transport (Fig. 9) [192, 193]. The intra and extracellular enzymes present in the endothelial cells work as a metabolic barrier against lipophilic substances [194].

#### Formulation of nanocarriers and its mechanism of delivery

Several nanocarriers including liposomes and solid lipid nanoparticles have been reported to deliver drugs across the BBB successfully. However, hexapeptide dalargin (Tyr-D-Ala-Gly-Phe-Leu-Arg), was the first drug delivered to the brain coated with polysorbate 80 nanoparticle [195]. The leucine-enkephalinanaloguedalargin was investigated as a model drug to study analgesic effects as well as its penetration across the BBB. Here, the dalargin bound to nanoparticles without polysorbate 80 demonstrated no analgesic effect. The most challenging research has focused on targeted drug delivery across the BBB to diagnose and treat various neurological disorders. Generally, the transport mechanism across the BBB can be categorized into three mechanisms: receptor-mediated, carrier-mediated, and vesicular transport [196]. Most nanosized systems use adsorptive-mediated transcytosis and receptor-mediated transcytosis as the two major mechanisms to deliver neurotherapeutics [197]. The negatively charged cerebral endothelial cells can be made to interact with nanoparticles by adding positive charges. This can be achieved by different procedures. The first method is to make a nanoparticle which bears positive charges at physiological pH 7.4. The second method makes use of surface functionalization of the nanoparticle with positively charged molecules thereby combining physicochemical features and biological activity. Cell-penetrating peptides like TAT-peptides (derived from HIV) and cationic proteins like albumin are widely used for nanoparticle anchoring that brings about the passage of drugs across the BBB [198].

Receptors for the uptake of different types of ligands (growth factors, enzymes and plasma proteins) are present in endothelial cells. For example, insulin molecules bind to its receptors present in specialized areas of the plasma membrane called coated pits. These coated pits invaginate into the cytoplasm, get separated from the cytoplasmic membrane and form coated vesicles. The ligand will dissociate from the receptor after acidification of the endosome [191]. The BBB expresses a variety of receptors including transferrin receptor (TfR), insulin receptor (IR), low density lipoprotein (LDL) receptor, diphtheria toxin receptor, nicotinic acetylcholine receptor (nAChR), scavenger receptors and class B type receptors. Hence, ligands can be used to decorate the deliver system that enables and enhances transport [199]. Qiao et al have successfully developed a brain delivery probe by covalently conjugating lactoferrin to the PEG-coated Fe_3_O_4_ nanoparticles to achieve receptor-mediated delivery of nanoparticles across the BBB [196]. In carrier mediated transcytosis, carriers mediate the transport mechanism. Carriers for glucose, amino acids, purine bases, nucleosides, and choline are present in endothelial cells and act as a transport system that can deliver the drug. Their main role is supplying nutrients to the brain. In addition, they serve as a transport carrier to deliver drugs. Liposome incorporated mannose derivatives were able to cross the BBB via a glucose transporter in mouse brain [200, 201].

#### Methods of BBB drug delivery

Approaches for delivery of drugs across the BBB can be broadly divided into the following categories: direct injection and implantation, chemical modifications, the temporary opening of the BBB using permeability enhancers and nano-enabled delivery platforms via the intravenous (IV) route and intranasal pathway. Several chemical agents circulating in the plasma membrane or secreted from cells can enhance BBB permeability. Some of the agents that weaken the BBB function include bradykinin, histamine, serotonin, glutamate, purine nucleotides, adenosine, platelet-activating factor (PAF), phospholipase A2 (PLA2), arachidonic acid, prostaglandins, leukotrienes, interleukins(IL-1α, IL-1β, IL-6), tumor necrosis factor-α (TNF-α), macrophage-inhibitory proteins MIP1 and MIP2, complement-derived polypeptide C3a-desArg, free radicals and nitric oxide, to name a few [192, 202].

The drug penetration of the BBB can be improved by special chemical modifications like lipidization and prodrug approach. Chemical modifications focus on the structural rearrangement of the drug to enhance their physicochemical properties. In lipidization, lipid molecules are added at the polar end of the drug molecule providing better permeability than the normal drug [202]. In the pro-drug approach, the drug is distinctively modified to enhance the capillary permeability. For that, the pro-moiety has to permeate through the membrane and once it reaches the brain, the conversion of pro-drug to the active parent drug will take place by enzyme catalysis [203]. Neural therapeutic agents can be delivered quickly within minutes by nasal administration. Lower molecular weight drugs having higher lipophilicity can easily enter the central nervous system. These drugs pass through the olfactory nerve where they first enter the respiratory epithelium followed by entry into the systemic circulation. This method delivers drugs into the deeper regions of the brain. The main limitation of this delivery system is that only small molecular weight drugs can be delivered efficiently. Some drugs cannot be internalized by the olfactory sensory neurons in the olfactory epithelium and hence cleared from the CNS easily. The nanoparticle drug delivery system overcomes this limitation and improves the persistence of the drug in the CNS. For example, chitosan modified molecules showed much longer residency time on the olfactory epithelium [204]. PEG-PLGA nanoparticle coated with odorranalectin that has low immunogenicity is widely used as a carrier for the nose to brain delivery. Lactoferrin conjugated PEG-PLGA nanoparticles and poly(ethylene glycol)-poly(ε-caprolactone) polymersomes conjugated with mouse anti-rat monoclonal antibody OX26 are also commonly used [200]. Due to mucociliary mechanisms the drugs get easily removed from the delivery site, reducing the contact period with nasal epithelium and delivery into the CNS following intranasal administration. To enhance the brain uptake and effective drug delivery, mucoadhesive in combination with microemulsion are used. To increase brain uptake and escape from clearance by P-gp mediated efflux, intranasal pretreatment with an inhibitor such as rifampin before intranasal administration of a P-gp substrate like verapamil is recommended [202, 205].

### Oral-route of administration

Oral delivery is the most common route of drug administration with high levels of patient acceptance. The oral route is the most preferred route for drug administration due to greater convenience, pain avoidance, efficacy, high patient compliance, and risk reduction of cross-infection and needle stick injuries [206]. It is expected to overcome the disadvantages associated with injection such as tissue injury, pain, adverse reactions, and poor patient compliance. However, the oral availability of the drug depends on the solubility and permeability of the compound [207]. Furthermore, oral delivery of peptides or proteins frequently suffers from the acidic environment and enzymatic system of the gastrointestinal tract (GIT) leading to the degradation of the protein thereby decreasing the therapeutic value. Therefore, several essential approaches have been tried to enhance the stability of the protein and peptide drugs and increase absorption [208, 209]. Site-specific delivery systems, chemical modification of peptides (e.g., lipophilic derivatives, synthesis of peptidomimetics, etc.), bioadhesive systems and concomitant administration of penetration enhancers or protease inhibitors have been investigated to improve the oral delivery of peptides [210]. After oral administration, the nanocarriers will encounter the physicochemical environment of the GIT. The human intestinal epithelium is composed of villi that increase the total absorptive surface area in the GIT to 300–400 m^2^ and acts as a physical barrier to drug absorption [138]. It is composed of absorptive enterocytes and for a large part sprinkled by mucus-producing goblet cells, endocrine, and Paneth cells. Immunocompetent cells (B and T lymphocytes, dendritic cells) are located in the lamina propria beneath the epithelium except for intraepithelial lymphocytes and dendritic cells that are inserted between the enterocytes. Biological fluids influence the strength of particles even before they enter and have contact with the intestinal cells.

#### Nanotechnology in oral-drug delivery

**Fig. 10:**
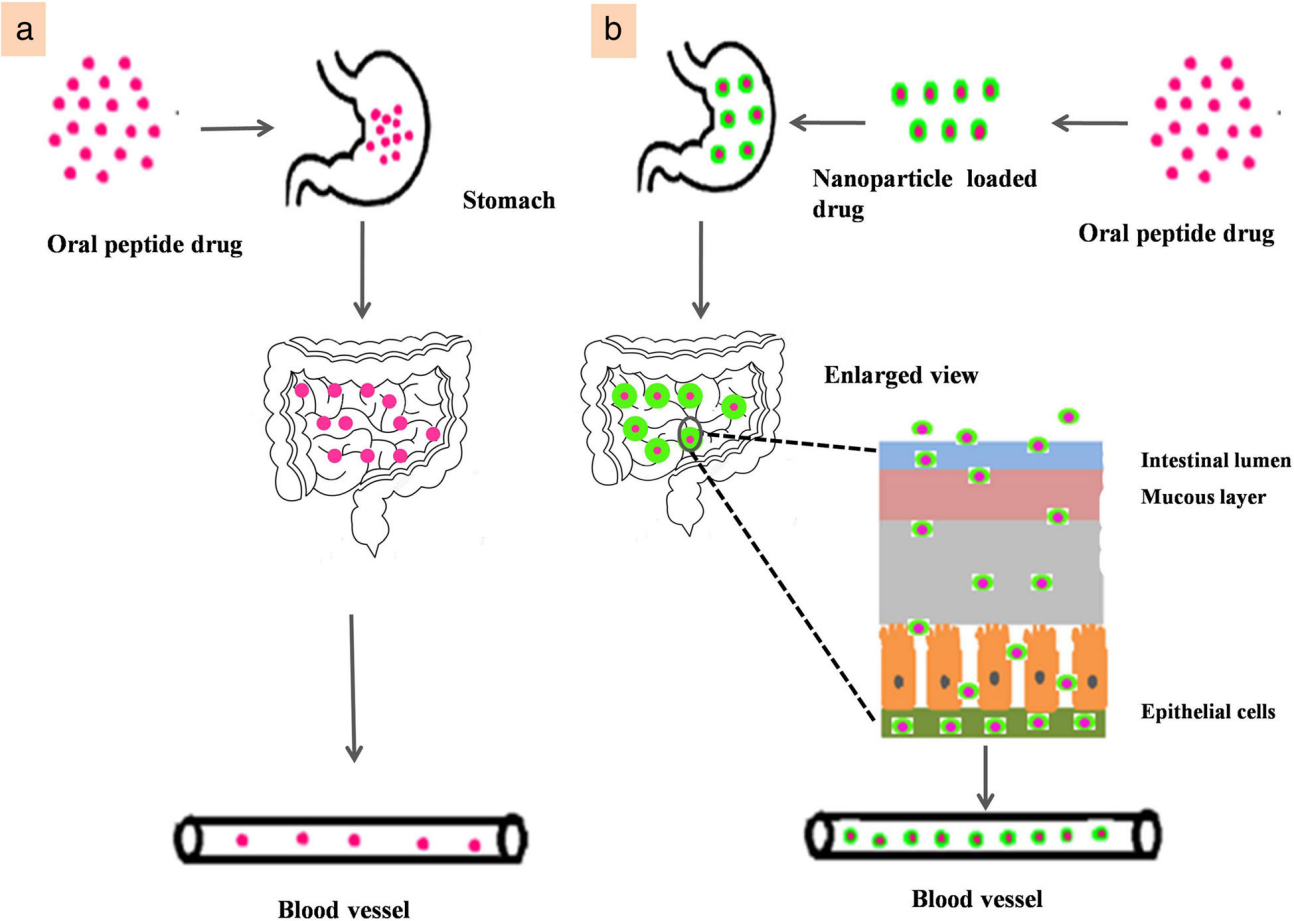
Administration of pH sensitive peptide drug via oral delivery. **a** The peptide drug administered orally degraded particularly in stomach due to proteolytic enzymes which result in poor availability of drugs. **b** The nanoparticles shields drugs and prevent from enzymatic degradation. Hence attains the efficient distribution of drugs

Nanotechnology comes with its own set of advantages in the drug delivery field, particularly in oral drug delivery. It allows the (i) delivery of poorly water-soluble drugs, (ii) targeting of drugs to a specific part of the gastrointestinal tract, (iii) transcytosis of drugs across the tight intestinal barrier and (iv) intracellular and transcellular delivery of large macromolecules [211]. Nanoparticle encapsulation is one such method to overcome the GI barrier, protect the drug from enzymatic degradation and release them in a controlled or systemic manner [212]. Use of a biodegradable polymeric nanoparticle is another promising approach to the pre-oral delivery of protein and peptide drugs with improved drug efficacy (Fig. 10) [213–215]. Polymeric nanocarriers can protect the drugs thereby increasing the absorption rate, and the nanocarrier composition will strongly influence its stability in the GIT. If nanoparticles are prepared with insoluble polymers, they will neither be degraded nor rapidly release the drug. In contrast, water-soluble polymers which form polyelectrolyte nanoparticles will be influenced by the pH or ionic strength and are more likely to be destabilized. Even if their kinetic stability is better than surfactant micelles, polymeric micelles concentration should remain above the critical micelle concentration upon dilution in the GIT to avoid release in the GIT and should be exposed to an ionic strength below their flocculation point [160].

Transport carriers and pro-drug approaches are also used for oral drug delivery and one prime example is transferrin as a carrier to deliver insulin. Polymeric micelles have been reported to cross the intestinal barrier after oral administration and therefore it is effectively used for oral drug delivery [216]. Zhang et al combined starch nanoparticles as the backbone and poly(L-glycolic acid) as a graft to develop a pH-responsive starch nanoparticles-g-PGA (SNP-g-PGA) that acted as a carrier to orally deliver insulin [217]. Considerable advancements are still required for the development of innovative materials and technologies to maximize drug absorption and stability in oral-drug delivery. Glucagon-like peptide-1 (GLP-1) is a small peptide hormone produced from intestinal L-cells and effective in lowering hyperglycemic conditions. Because of very short plasma half-life (< 5 min) and rapid metabolic clearance, the anti-diabetic effect of GLP-1 could be better utilized with oral-gene delivery methods. In this context, antibody-mediated (human IgG1 (hIgG1)-Fc-Arg/pDNA complexes were prepared as an oral-gene delivery system for the prevention of type 2 diabetes mellitus (T2DM) [218].

Oral delivery of methylthioadenosine (MTA) to the brain by solid lipid nanoparticles was reported for the effective management of multiple sclerosis-like conditions in mice. As compared to plain MTA, MTA loaded SLN not only offers high drug entrapment but also increased the half-life of MTA from 28 min to 1.25 h and improve the locomotors activity from 49 to 79%, respectively [219]. However, recent advancements in oral-drug delivery include the development of bioadhesive food protein nanoparticles using zein (Z) and whey protein (WP). The hydrophobic corn protein zein is used as a core and whey protein acts as a shell to deliver the antiretroviral drug Lopinavir (LPN) and fenretinide, an investigational anticancer agent. Similar to MTA loaded SLN, ZWP nanoparticles have also increased the half-life and bioavailability of both drugs when administrated orally [220]. Oral-administration routes continued to improve the therapeutic effect of peptides. However, challenges associated with antihypertensive peptides are rapid degradation and poor bioavailability. Though injections are considered an alternate routine of peptide drug administration, it results in poor patient compliance because of repeated injections. Hence, a novel oral peptide delivery system like Tyr-Gly-Leu-Phe (YF4)-loaded lipid nanoparticles (YF4-LNPs) was developed to utilize the advantage of both polymer nanoparticles and liposome. The in vitro release profile showed burst release of 80% free YF4 within 6 h while YF4-LNP showed less than 40% release in 24 h. The in vivo antihypertensive activity in the animal model showed the decrease of SBP (Systolic Blood Pressure) by 15.6 mmHg at 4 h post-administration while in YF4-LNPs, blood pressure decreased by 43.5 mmHg in about 2 h post-administration [221].

### Inhalation route

Pulmonary delivery has several irreplaceable advantages over other delivery routes such as oral or injection. It avoids first-pass hepatic metabolism thus reducing dose requirement and side effects. Pulmonary delivery also allows local delivery of therapeutics targeting respiratory diseases such as asthma, chronic obstructive pulmonary disease (COPD), and cystic fibrosis. The pulmonary route offers other advantages such as a high surface area with rapid absorption due to high vascularization and circumvention of the first-pass effect [222]. The pulmonary route has been used for local delivery of drugs like antibiotics (cyclosporine, tobramycin, amikacin, fluoroquinolones) [223] proteins and peptides (insulin, amylin, calcitonin) [224], chemotherapeutics (doxorubicin, fluorouracil, cisplatin) [225, 226], interferon (interferon-α, interferon-γ etc) [223], and vaccines (measles, influenza, tuberculosis, hepatitis) [227, 228].

The lung consists of two functional parts, the airways (trachea, bronchi, and bronchioles) and the alveoli (gas exchange areas). The conducting zone consists of the first 16 generations of airways comprised of the trachea (generation 0), which separates into the two mainstem bronchi and subdivides progressively into smaller bronchi and bronchioles. The respiratory zone consists of all structures that participate in gas exchange and begins with the respiratory bronchioles [229]. The particles that are less than 5–6 μm are deposited into the trachea-bronchial region. Ultrafine particles (1–2 μm) settle in the bronchioles and particles at the nanoscale (< 1 μm) are delivered into the lower respiratory system. Ultra-small-sized nanoparticles such as dendrimers (< 20 nm) showed efficient delivery to the alveoli but they often presented low retention in the lungs due to the rapid penetration into the bloodstream [230–232]. The most important mechanisms of particle deposition in the respiratory tract are inertial impaction, gravitational sedimentation, and diffusion (Brownian motion).

#### Nanoparticle formulations for drug delivery to the lungs

**Fig. 11:**
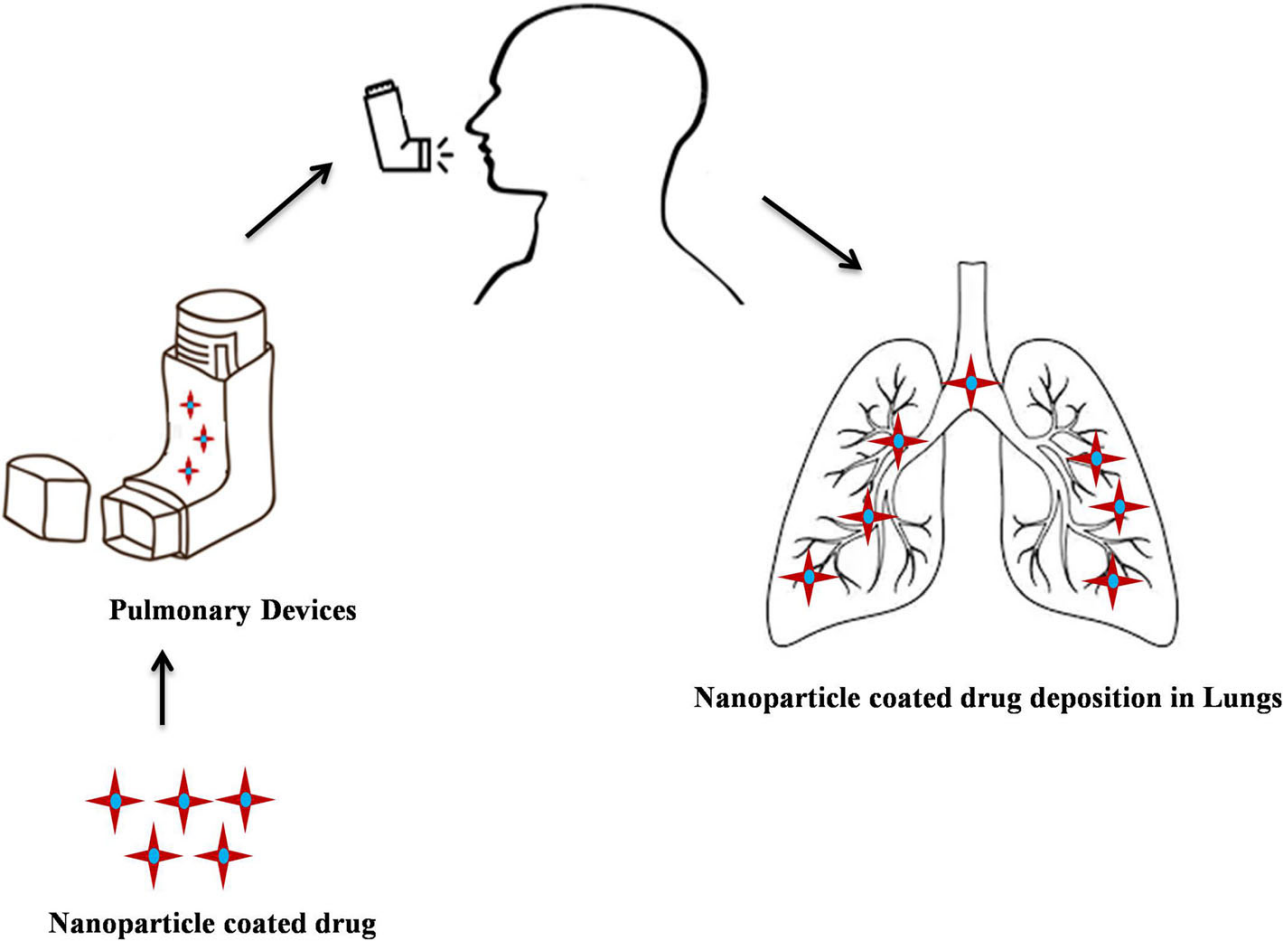
Pulmonary drug delivery via inhalation. The concept of nanoparticle incorporated drugs for pulmonary delivery is, when it is inhaled it will pass through oropharynx and deposited in alveoli of lungs with the help of suitable inhalation devices. The pulmonary device containing nanoparticle coated drugs, when inhaled will pass through oropharynx and deposited in the alveoli of lungs. Subsequently, the nanoparticle coated drug aids in sustained release of drugs from the lungs and thus improved distribution in systemic circulation. It offers high surface area with rapid absorption vascularization and circumvention of the first pass effect

A challenge for nanoparticulate drug delivery to the lungs is to understand the fate of the particles and their interactions with biological systems. To successfully deliver an inhalable drug it should overcome pulmonary clearance (mucociliary escalator, alveoli) and detoxification activity of enzymes like cytochrome P450. Rapid particle clearance reduces sustained delivery of the drug and particle translocation might bring nanoparticles to undesired areas of the body. To overcome these obstacles and increase efficiency, a particulate based drug delivery system is introduced. It uses carriers (liposomes, solid lipid nanoparticles, polymers, etc.) to encapsulate the drug thereby increasing half-life of the drugs [233]. Nanoparticles could provide the advantage of sustained release in the lung tissue, followed by the systemic circulation leading to a reduction in dosage frequency and improved patient compliance (Fig. 11). Nanoparticle deposition in the respiratory tract is determined predominantly by diffusional alteration due to the thermal motion of air molecules interacting with particles in the inhaled and exhaled air streams [234, 235]. Three types of pulmonary delivery devices are commercially available: 1) pressurized metered-dose inhalers (pMDI), 2) nebulizers and 3) dry powder inhalers (DPI). DPI and MDI make use of impaction were aerosol particles travel at high velocity settling in the oropharynx region due to centrifugal force. Sedimentation is the most important technique for the nanoparticulate system since the particle settles for a long time at the site and, as a result, increases the efficiency of the drug [222]. The aerodynamic diameter of nanoparticles is the primary determinant for in vivo distribution of the inhaled nanoparticles [236]. Depending on the particle size, shape and ventilation parameters deposition occur in all regions of the lungs (the airways and the alveoli). With decreasing particle diameter below about 500 nm, the deposition increases in all regions of the lung because of the increasing diffusional mobility [237].

The pharmacokinetics of the nanoparticles can be altered after a structural modification. The dendrimers without any surface modification get absorbed into the bloodstream with limited lung retention, but PEG-modified dendrimers with larger sizes (> 78 kDa) will accumulate in the lungs [238]. To increase the shelf life of the drugs, they can be coated with stealth material (e.g. hyaluronic acid) which forms a hydration layer that prevents immune recognition [239]. Rifampicin is a known first-line drug for tuberculosis that exhibits self-aggregation in the aqueous phase which affects the preparation of liquid pediatric tuberculosis formulation. The self-aggregation of drug molecules is resolved by encapsulating within the commercial polymeric micelles Kolliphor® HS 15. The nanoscale Kolliphor® HS 15 micelles have improved the aqueous solubility and microbicidal activity to 14.3 fold and 2.5 fold, respectively [240]. Anti-inflammatory drug, budesonide encapsulated in solid lipid nanoparticle suspension (SLNPs) was used to test the efficacy of endotracheal aerosolization (ETA) device for pulmonary delivery. In ETA, nanoparticle suspensions are directly aerosolized within the trachea and readily deposited into the pulmonary region. Furthermore, it is a non-invasive and promising method with high efficiency. The budesonide loaded SLNPs formulation has shown 80% pulmonary deposition in Sprague–Dawley rats and a high in vitro emission rate [241]. Similarly, pulmonary delivery of nanocomposite microparticles (NCMPs) i.e. PGA-co-PDL nanoparticles with microRNA (miR-146a) by dry powder inhalation was useful for the treatment and management of chronic obstructive pulmonary disease (COPD) [242]. The activity of miR-146a was preserved after the spray-drying process and miR-146a loaded NCMPs were used to silence the target genes IRAK1 and TRAF6. MiR-146a-5p demonstrated its protective effects against tumorigenesis and development of diverse neoplasms, including non-small cell lung cancer (NSCLC) by down-regulating the IRAK1 (IL-1 receptor-associated kinase 1) and TRAF6 (TNF receptor-associated factor 6) expression [243].

### Intravenous delivery

**Fig. 12:**
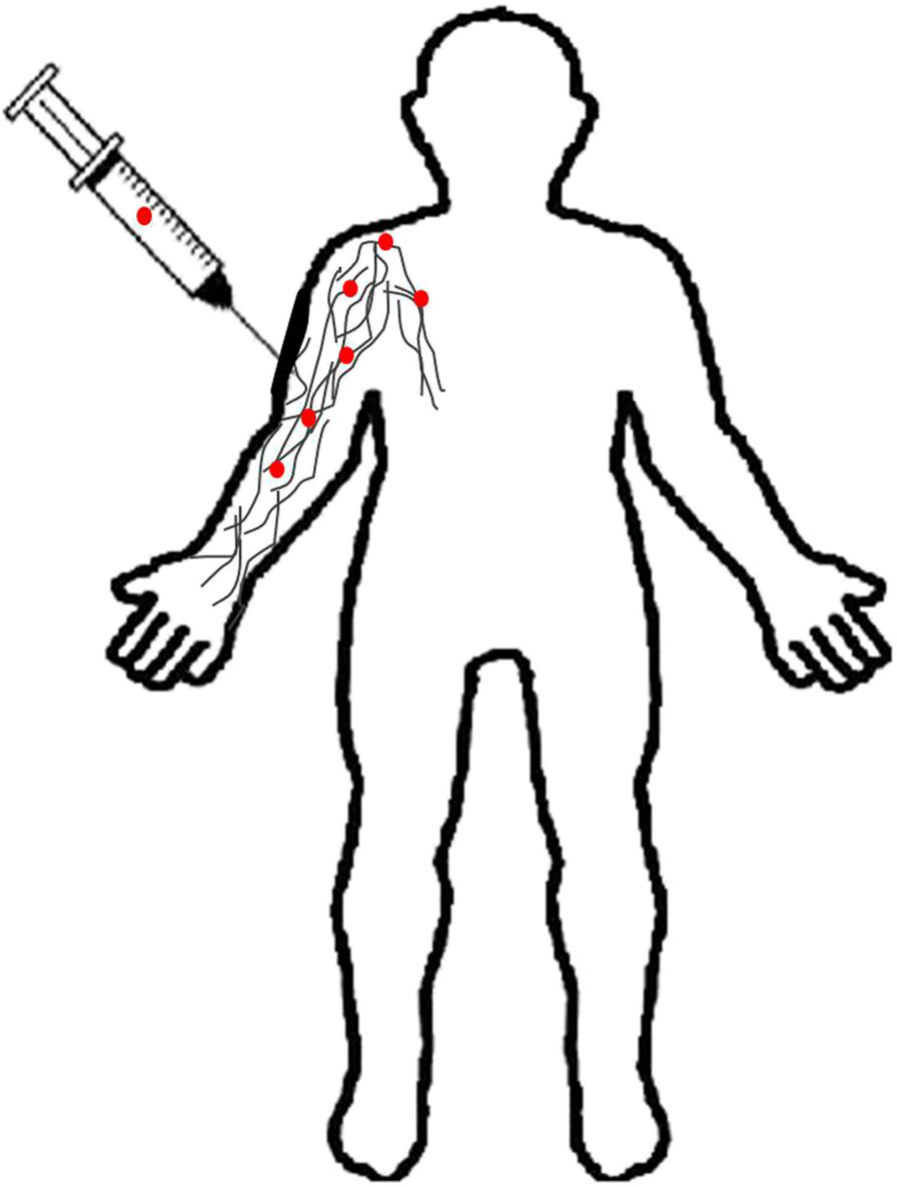
Systemic delivery of nanoparticles by intravenous injection. Intravenous drug administration via blood stream is equally popular route of drug administration and offers the systemic action as well as complete bioavailability. The uncoated or raw nanoparticles have often suffers with the effect of opsonization or macrophage uptake, especially nanoparticles with <∼5 nm rapidly undergo renal clearance upon intravenous administration. Surface tailoring is the effective way of prevent clearance and improve the cellular uptake for maximum drug accumulation in tumor sites. Nanoemulsions and micellar nanocomplex are significantly used in recent times to enhance the anti-tumor effect of the drug with infinitesimal off-target toxicity

Nanoparticles can be administered through different routes including intravenous and intraperitoneal injection, oral administration, and pulmonary inhalation. The IV route provides almost instantaneous response and allows wide-ranging control of the rate of drug contribution into the body. It is also suitable for drugs which cannot be absorbed by the gastrointestinal tract or which cannot be injected into muscles or other tissues, equally important it overcomes the problem of first-pass metabolism [244]. Expensive drugs such as peptides and proteins are delivered efficiently by intravenous route. Intravenous administration overcomes the degradation by proteolytic enzymes (Fig. 12). The main advantage of intravenous drug delivery is the rapid onset of action and complete bioavailability of drugs even with low doses. There are many risks associated with IV route because of the direct exposure of the drug in the systemic circulation. It is painful for the patient, expensive and requires the assistance of experienced healthcare personnel. The first intravenously administered nanoparticulate product, Abraxane® (a reformulation of paclitaxel), was approved by the FDA in 2006 [245, 246].

The major difficulties in current cancer therapy are mostly the drug side effects due to drug accumulation, cancer recurrence, and delay in disease stabilization. These challenges can be overcome by nanomedicines. Clinical trials in humans demonstrated that controlled release nanocarriers can be intravenously infused and guided towards local tumor site that in turn augment the efficacy of solid tumors treatment. It reduces the toxic side effects of the drugs and produces prolonged remission. Drug polymer conjugates and nanoemulsions are mainly explored for targeting prostate cancer [247]. Paclitaxel is a first-line chemotherapy drug which is commercially available as paclitaxel-cremophor (1:1) combination. Paclitaxel, when administered with cholesterol-rich nanoemulsion (LDE), had displayed low toxicity and increased anticancer activity in a mouse model. Further, LDE tends to concentrate on solid tumors and binds to cancer cells overexpressing LDL receptors. Recently, the pharmacokinetic and tumor uptake efficiency of paclitaxel-LDE and paclitaxel-cremophor was compared in human gynecological cancers. The mean half-life of paclitaxel-cremophor were 6.62 ± 2.05 h whereas paclitaxel-LDE has shown T1/2 of 14.51 ± 3.23 h, and it also showed higher targeting in tumor tissues (3.5times) than normal tissues [248].

At present, various studies have been conducted on the delivery of nanoparticle-associated drugs by the intravenous route. The nanoparticle is not able to efficiently deliver drugs due to RES uptake. To overcome this problem, surface modification of nanoparticles can be carried out. Xiang et al developed SLNP-containing dexamethasone acetate (DXM). DXM alone and DXM-SLNP are intravenously administered to mice. For DXM-SLNP and DXM, the biodistribution showed a significantly different pattern. The area under the drug concentration-time curve of DXM-SLNP in the lung was 17.8-fold larger in comparison to that of DXM solution alone [249]. For the intravenous application of emulsions, the size of oil droplet should be below the size of the smallest blood vessel in the lungs which is 5 μm. The mean droplet size of these particles is in the range of 200–400 nm and is consequently called nanoemulsion [250]. Though iron has been used to treat anemia for more than 300 years, oral iron therapy invariably results in gastrointestinal toxicity and takes a long time to combat the disease.

Intravenous delivery overcomes this limitation to some extent with fewer side effects and rapid release of iron. Third generation IV-iron therapies have especially improved the efficacy significantly without any toxicity issues encountered during the old-generation iron therapy. The accelerated dose of Cosmofer (iron dextran) administration has proven to be effective, very safe, time-saving and it enhances the reduction in nursing time without any late adverse reaction for the chronic kidney disease (CKD) patients [251]. As of now, the best-developed IV formulation comprises iron–oxyhydroxide core encompassed within carbohydrate shells of different sizes and polysaccharide branches. However, the toxicity profiles should be evaluated because long-term clinical use is widespread [252]. A new micellar nanocomplex consisting of IONP conjugated HA was fabricated to deliver the drug homocamptothecin (HCPT) via intravenous administration. The combined magnetic and CD44 binding ability from IONP and HA, respectively have ensured increased uptake and theranostic potency of HA-IONP/HCPT (HIH) in human squamous cell carcinoma cell line (SCC-7 cells) through superior EPR permeability retention targeting. The administration of 3 mg/kg of HIH in the presence of a magnetic field showed complete disappearance of the tumor after 14 days in mice model. The results demonstrated the translational potential of HIH nanocomplex for cancer theranostics owing to its excellent tumor targeting ablation with no systemic toxicity [253].

## Nanotoxicity

Nanoparticle drug delivery offers enormous benefits due to it highly stable nature and its ability to encapsulate both hydrophilic and hydrophobic substances. Importantly, nanoparticles are consistent with various routes of administration [156]. Addressing nanoparticle drug delivery nanotoxicity is of great significance. As of now (July 2019) PubMed has enlisted 43,570 and 21,835 articles for the search terms “nanoparticle drug delivery” and “nanoparticle toxicity”, respectively. The unique properties of nanoparticles such as the small surface to volume ratio are alluring and possibly valuable from an engineering or biomedical point of view. Likewise, the properties that may give rise to unexpected toxicities are equally interesting [254]. The toxicity level of anionic nanoparticles are considerably less toxic; whereas the cationic nanoparticles like gold and polystyrene nanoparticles have been reported to cause hemolysis and clotting [110].

Nanomaterials can enter the body through several routes including the skin, respiratory tract, parenteral administration, etc. In the blood, it will come in contact with plasma proteins that will probably lead to the formation of protein corona which may modify the pharmacological properties of the nanoparticles. The interaction between the nanoparticle and the body should be properly assessed since toxicity is of great concern [255]. In vivo and in vitro studies of nanoparticles have shown that the minor toxicities observed are due to increasing ROS levels and disruption of the host homeostasis [256]. The ROS could further damage the genome and create oxidative stress conditions that in turn induce micronuclei formation. Irrespective of their size, amorphous TiO_2_ (30 nm) and silver nanoparticles (15 nm) induce the highest generation of reactive oxygen species. The possible engulfment of silver nanoparticles and quantum dots by macrophages certainly will enhance the expression of inflammatory mediators TNF-α, MIP-2 and IL-1β, irrespective of their size [257]. Nanoparticles tend to accumulate in the liver, therefore the detailed mechanism of how these particles are eliminated from the body should be investigated [156]. A single and multi-walled carbon nanotube induces platelet aggregation whereas their building blocks C-60 fullerenes do not. The principle behind nanomaterial functioning will be addressed in detail for successful and safe drug delivery. The use of nanoparticles is increasing. Similarly, toxicity issues must also be considered.

## Conclusions

This analysis provides an overview of the different nanocarriers/NPs and various routes of drug administration for improved drug delivery along with detailing the challenges associated with the nanocarrier systems. With the help of cutting-edge technology, a variety of natural and synthetic polymers have been successfully engineered to deliver drugs with improved efficiency. Though nanoparticles offer higher drug loading, better bioavailability, etc., nanoparticle-mediated toxicity is yet to be resolved to satisfaction. Hence, extensive research and development is currently focused on initiating controlled drug delivery with less toxicity. Polymers like chitosan are commonly used for drug delivery owing to their biodegradable, biocompatible and mucoadhesive properties. For the past decennium, the concept of biomimetic has been introduced in material design to create more biologically attractive nanocarriers. This could either be achieved by introducing suitable ligands to the CNT surface or by fabricating chitosan nanoparticle with the desired chemical molecule or moiety promoting self-assembly for increased cellular uptake. The successful delivery of a drug to the target region requires not only an ideal nanocarrier but also an effective route of drug administration that enables crossing the blood-brain barrier. However, each route of administration has its advantages and disadvantages when it comes to targeted drug delivery. To overcome the limitations of different administration routes, superior understanding of intercellular, transcellular and other carrier-mediated transporting pathways are essential to develop the next-generation of futuristic nanocarriers. The creation of such an advanced nanotherapeutic system will mark the beginning of a new era in nanotechnology-based drug delivery.

## Data Availability

This is a review article. All data generated or analyzed during this study are included in this published article.

## Abbreviations

5-ASA: 5-Aminosalicylic acid
5-FU: 5-Fluorouracil
AA: Acrylic acid
AJs: Adherens Junctions
ALL: Acute Lymphoblastic Leukemia
AUC: Area under the Curve
AZ-HECTS: Azidehydroxyethyl chitosan
BBB: Blood-brain barrier
BML: Bubble-generating magnetic liposomal drug delivery system
BSA: Serum Albumin
CKD: Chronic Kidney Disease
CLSM: Confocal Laser Scanning Microscopy
CNS: Central Nervous System
CNT: Carbon Nanotubes
COPD: Chronic Obstructive Pulmonary Disease
CQDs: Carbon Quantum Dots
CT: Computed Tomography
DDS: Drug Delivery System
DMSA: Dimercaptosuccinic acid
DOX: Doxorubucin
DPI: Dry Power Inhalers
DXM: Dexamethasone acetate
EA: Ellagic acid
EMA: European Medicine Agency
EPR: Enhanced Permeability and Retention
ETA: Endotracheal aerosolization
FA: Folic acid
FAE: Follicle associated epithelium
FDA: US Food and Drug Administration
GALT: Gut Associated Lymphoid Tissue
GIT: Gastrointestinal tract
GLP-1: Glucagon like peptide
GMO-MNPs: Glycerol mono-oleate-coated magnetic nanoparticles
HA: Hyaluronic acid
HC: Hydroxyethylacryl Chitosan
HCPT: 10-hydroxy camptothecin/ homocamptothecin
HER2: Human epidermal growth factor receptor 2
HIH: HA-IONP/HCPT
IG: Indocyanine green
IL: Interleukin
IONPs: Iron Oxide Nanoparticle
IR: Insulin Receptor
LDE: Cholesterol-rich nanoemulsion
LDL: Low Density Lipoprotein
LMWC: Low Molecular Weight Chitosan
LPN: Lopinavir
MDR: Multidrug Resistance
MIP: Macrophage-Inhibitory Protein
MMP: Matrix Metalloproteinases
MNPs: Magnetic nanoparticles
MPS: Mononuclear Phagocyte System
MRI: Magnetic Resonance Imaging
MTA: Methylthioadensine
MW: Molecular Weight
MWCNT: Multi-wall Carbon Nanotubes
nAChR: nicotinic Acetylcholine Receptor
NC: Nanocarriers
NIR: Near-infrared
NPs: Nanoparticles
OSC: Organic Solar Cells
PAF: Platelet-activating factor
PAMAM: Polyamidoamine
PCL: Polycaprolactones
PDT: Photodynamic therapy
PE: Phosphoethanolamine
PEG: Polyethylene glycol
PEGMEA: PEG methyl-ether acrylate
PEI: Polyethyleneimine
PET: PositronEmission Tomography
P-gp: P-glycoprotein
PLA: Polylactic acid
PLA2: Phospholipase A2
PLGA: Poly(lactic-co-glycolic acid)
PLL: Poly(L-lysine)
pMDI: pressurized Metered Dose Inhalers
PMMA: Poly(methyl methacrylate)
pNIPA: poly(N-isopropyl acrylamide)
PPI: Poly(propylene imine)
PTT: Photothermal therapy
PTX: Paclitaxel
QDs: Quantum Dots
rdAds: replication-defective adenoviruses
RES: Reticuloendothelial system
ROS: Reactive Oxygen Species
SA: Sodium Alginate
SBP: Systolic blood pressure
SLNP: Solid Lipid Nanoparticles
SMANCS: Styrene maleic anhydride-neocarzinostatin
SNP-g-PGA: Starch nanoparticles-poly(L-Glycolic acid)
SPM: Superparamagnetic
SPR: Surface Plasmon Resonance
SWCNT: Single Wall Carbon Nanotubes
TDD: Transdermal Drug Delivery
TfR: Transferrin Receptor
TJs: Tight Junctions
TMC: Trimethyl Chitosan
TNF-α: Tumor Necrosis Factor-α
W: Whey protein
Z: Zein
ZWP: Zein whey protein
